# A Sensitive and Wide Coverage Ambient Mass Spectrometry Imaging Method for Functional Metabolites Based Molecular Histology

**DOI:** 10.1002/advs.201800250

**Published:** 2018-10-07

**Authors:** Jiuming He, Chenglong Sun, Tiegang Li, Zhigang Luo, Luojiao Huang, Xiaowei Song, Xin Li, Zeper Abliz

**Affiliations:** ^1^ State Key Laboratory of Bioactive Substance and Function of Natural Medicines Institute of Materia Medica Chinese Academy of Medical Sciences and Peking Union Medical College Beijing 100050 P. R. China; ^2^ Center for Imaging and Systems Biology Minzu University of China Beijing 100081 P. R. China

**Keywords:** functional metabolites, mass spectrometry imaging, molecular histology, sensitive and wide coverage

## Abstract

Histological examination with a deep link between functional metabolites and tissue structure and biofunctions will provide important in situ biochemical information, and then essentially reveal what has happened in tissue at the molecular level. However, due to the complexity and heterogeneity of tissue samples and the large number of metabolites, it is still a challenge to globally map the diverse metabolites, especially for those low‐abundance functional ones. Here, a sensitive air flow‐assisted desorption electrospray ionization mass spectrometry imaging method for the mapping of a broad range of metabolites is presented. It exhibits properties characteristic of wide coverage, high sensitivity, wide dynamic range, rapid analysis procedure, and high specificity for tissue metabolites imaging. More than 1500 metabolites, including cholines, polyamines, amino acids, carnitines, nucleosides, nucleotides, nitrogen bases, organic acids, carbohydrates, cholesterol sulfate, cholic acid, lipids, etc., can be visualized in an untargeted analysis. The distribution of metabolites shows good spatial match with tissue histological structure and biofunctions in heterogeneous rat kidney, rat brain, and human esophageal cancer tissue. This method possesses the ability to globally showcase the molecular processes in tissue, and provide an insightful way for structural and functional molecular recognition in histological examination, even for intraoperative decision‐making.

## 1 Introduction

Global profiling of functional metabolites with spatial distribution in biotissue is key for understanding the molecular processes in biosystem, providing an insightful way for tissue‐specific molecular histology and pathology.^1^ However, the fact that over 41 000 metabolites of different classes appear in the extremely complex organism with significant content variation has made its detection difficult.^2^ The core of the problem lies in developing an untargeted, high sensitive, wide coverage, and high chemical specific imaging method, visualizing the spatial distribution of numerous metabolites in their native state, especially for those with extensive biofunctions but low‐abundance in tissue. Linking the spatial and temporal change of functional metabolites to tissue structure and biofunctions will essentially reveal what has happened in tissue at the molecular level.

**Figure 1:**
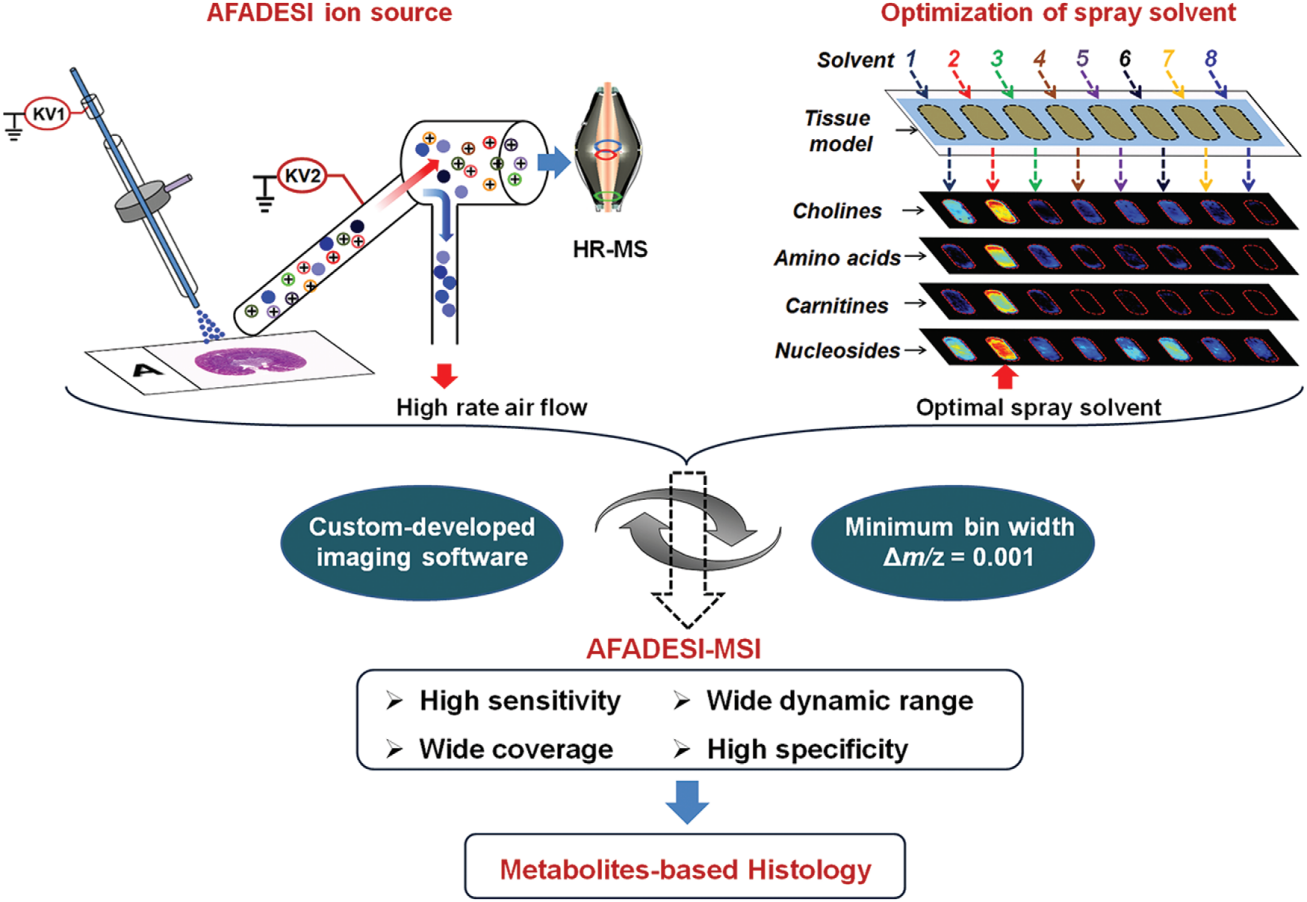
The strategy to develop a sensitive and wide coverage AFADESI‐MSI method for functional metabolites based molecular histology.

Mass spectrometry imaging (MSI) has the ability to simultaneously illustrate the relative abundance and present the spatial distributions of known or unknown biomolecules in tissue.^3^ Secondary ion mass spectrometry (SIMS) and matrix‐assisted laser desorption ionization (MALDI) are two important MSI methods which always be performed in vacuum.^4^ Atmospheric pressure MALDI‐MSI shows its ability for tissue imaging with sub‐micrometer resolution.^5^ Ambient ionization mass spectrometry techniques, such as desorption electrospray ionization (DESI) and laser ablation electrospray ionization (LAESI), have been developed for direct tissue imaging.^6^ Recently, an automated and biocompatible handheld mass spectrometry device, named MasSpec Pen was developed for rapid and nondestructive cancer diagnosis.^7^ Air flow‐assisted desorption electrospray ionization mass spectrometry imaging (AFADESI‐MSI) was developed by our group and applied in whole body molecular imaging, drug molecular mechanism study, and in situ biomarker discovery.^8^ For now, ambient ionization MSI has been widely used in histologic diagnosis of cancer and other diseases mainly based on lipid profiles.^9^ Several MSI studies have been reported to visualize small molecule metabolites in biosamples.^10^ However, given the complexity and heterogeneity of tissue sample, it is still challenging to develop a wide metabolites coverage method with high sensitivity, broad dynamic range, and high specificity. Here, we developed a rapid, sensitive, and wide coverage AFADESI‐MSI method to visualize metabolites' spatial distribution on tissue. The strategy is shown in **Figure** **1**. In this contribution, high‐rate air flow was introduced in custom‐built AFADESI ion source to enhance the in situ collection and sampling of droplets, thereby improving the sensitivity. Tissue homogenate models were constructed to optimize spray solvent for the detection of metabolites. High mass resolution orbitrap mass spectrometer coupled with custom‐developed highly discriminating imaging software MassImager (minimum bin width, Δ*m*/*z* = 0.001) ensures a precise assignment for metabolites with close *m*/*z*. As a result, thousands of metabolites and lipids were visualized in heterogeneous rat brain, rat kidney, and human esophageal cancer tissue from an untargeted analysis, providing robust structural and functional references for metabolites based molecular histology. The method exhibits properties characteristic of wide coverage, high sensitivity, and high specificity, making it particularly useful for tissue structural and functional molecular recognition.

## 2 Results

### 2.1 Wide Coverage, High Sensitivity, Wide Dynamic Range, and High Specificity of AFADESI‐MSI

**Figure 2:**
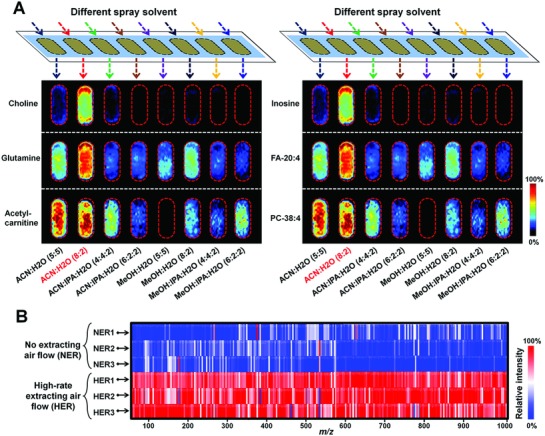
A) MS images of representative metabolites in tissue homogenate model under different spray solvent systems. B) Relative ion intensity of endogenous ions (*m/z* 70–1000) with no extracting air flow (NER) or with high‐rate extracting air flow (HER).

A home‐built AFADESI ion source was successfully coupled with high mass resolution Q‐Orbitrap mass spectrometer. Uniform and repeatable tissue models were designed to optimize spray solvent system for high sensitive and wide coverage metabolite analysis. The detailed process to prepare tissue homogenate model is given in Figure S3 (Supporting Information). Spray solvent ACN/H_2_O (5:5), ACN/H_2_O (8:2), ACN/IPA/H_2_O (4:4:2), ACN/IPA/H_2_O (6:2:2), MeOH/H_2_O (5:5), MeOH/H_2_O (8:2), MeOH/IPA/H_2_O (4:4:2), and MeOH/IPA/H_2_O (6:2:2) were successively tried to seek the optimal spray solvent system for different metabolites. The specific results of metabolites numbers, ion intensities, etc., are shown in the Supporting Information. MS images of representative endogenous metabolites in different spray solvent systems are shown in **Figure** **2**A. Finally, spray solvent system ACN/H_2_O (8:2) was chosen for its ability to dissolve and transport most metabolites. Moreover, high‐rate extracting air flow significantly increase the ion intensities of metabolites (Figure 2B). Under the optimized AFADESI‐MSI system, more than 1500 metabolites were detected after deducting background and isotope ions. These metabolites including cholines, polyamines, amino acids, carnitines, nucleosides, nucleotides, nitrogen bases, organic acids, carbohydrates, cholesterol sulfate, cholic acid, lipids, and other small molecule metabolites (Tables S1 and S2, Supporting Information).

**Figure 3:**
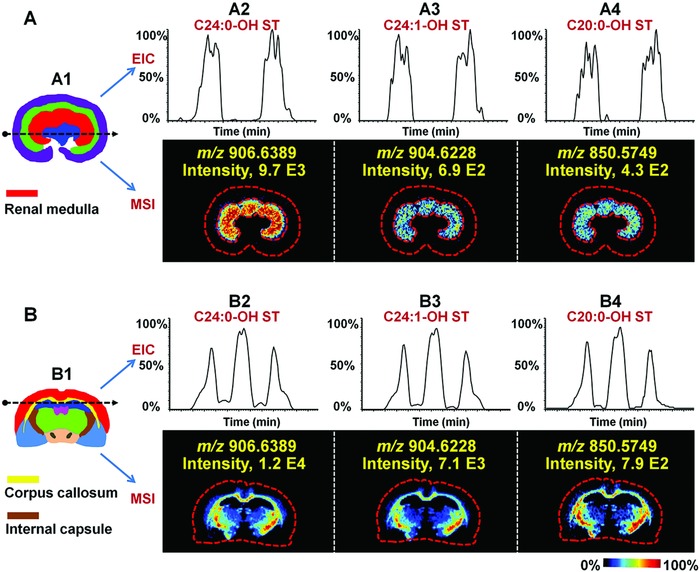
Extracted ion chromatography (EIC) profiles and MS images of ST. A1) The structure diagram of rat kidney. A2–A4) EIC profile and MS images of ST in rat kidney. B1) The structure diagram of rat brain. B2–B4) EIC profile and MS images of ST in rat brain.

For AFADESI, high‐rate air flow was introduced to enhance the in situ collection and sampling of droplets. This helps to improve the sensitivity and then expands the scope of metabolite profiling. For example, sulfatides (STs) play important roles in brain and renal diseases. As shown in **Figure** **3**, ST almost just located in renal medulla with a high signal‐to‐noise ratio (Figure 3A). The ion intensity ratio of C24:0‐OH‐ST (Figure 3A2) to C24:1‐OH‐ST (Figure 3A3) was consistent with previous quantitative LC‐MS/MS study.^11^ As for low‐abundance C20:0‐OH‐ST (Figure 3A4), though the ion intensity is 4.3E^2^, a renal medulla‐specific distribution was still clearly presented. In rat brain, STs were primarily found in corpus callosum and internal capsule (Figure 3B). What is more meaningful is that the low‐abundance C20:0‐OH‐ST (Figure 3B4), which has been identified as being unique to kidney and undetected in brain by LC‐MS/MS,^11^ still demonstrated a region‐specific distribution. To our knowledge, this is the first report on the detection of low‐abundance C20:0‐OH‐ST by MSI. Other typical STs in the kidney and brain are shown in Figures S11 and S12 (Supporting Information). These further illustrate that the high sensitivity of AFADESI‐MSI guarantee wide coverage detection.

**Figure 4:**
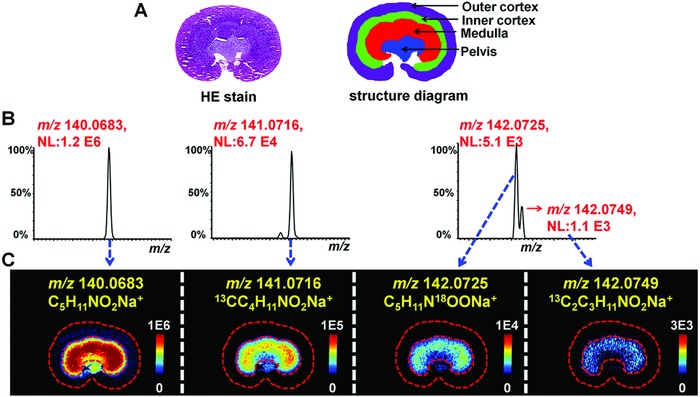
MS spectra and images of betaine and its isotope ions in rat kidney. A) HE stain and structure diagram of rat kidney. B) MS spectra of betaine and its isotope ions in rat kidney. C) MS images of betaine and its isotope ions in rat kidney.

Over three orders of magnitude dynamic range were achieved in functional metabolites based histological examination by using untargeted AFADESI‐MSI method. **Figure** **4** suggests that betaine (M, C_5_H_11_NO_2_) is mainly located in kidney medulla. The ion intensities of its [M+Na]^+^ ion (*m*/*z* 140.0683), its ^13^C isotope ion [M+1+Na]^+^ (*m*/*z* 141.0716), and its ^18^O and 2^13^C isotope ions [M+2+Na]^+^ (*m*/*z* 142.0725 and *m*/*z* 142.0749) are 1.2E^6^, 6.7E^4^, 5.1E^3^, and 1.1E^3^, respectively (Figure 4B). Although the intensity ratio of (*m*/*z* 140.0683)/(*m*/*z* 142.0749) > 1.0E^3^, they still demonstrated a clear and consistent medulla‐specific distribution (Figure 4C). Similarly, the MS images of choline [M]^+^ and its isotope ions showed a clearly uniform distribution in kidney (Figure S15, Supporting Information). Moreover, the dynamic range was further evaluated by analyzing tissue homogenate models with different concentration levels of berberine and D_9_‐choline (Figure S17, Supporting Information). The results show that the linearity range is 0.06–87.5 ng mm^−2^ (1458 folds) for berberine and 0.4–875 ng mm^−2^ (2188 folds) for D_9_‐choline. The wide dynamic range helps to display ion images with richer color and better layering and therefore giving a precise metabolites distribution in tissue.

**Figure 5:**
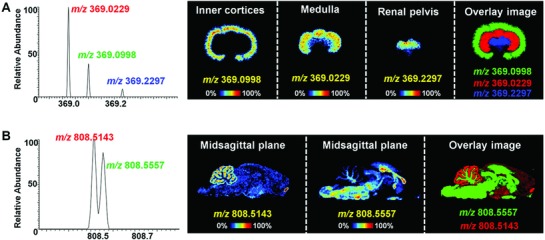
MS spectra and images of metabolites with close *m*/*z* in rat kidney and brain. A) MS spectra and images of *m*/*z* 369.0229, *m*/*z* 369.0998, *m*/*z* 369.2297 ions in rat kidney. B) MS spectra and images of *m*/*z* 808.5143, *m*/*z* 369.5557 ions in rat brain.

In heterogeneous tissue organs like brain and kidney, metabolites may present an entirely different spatial distribution at different microregion. However, the spatial distribution of low‐abundance metabolites may be masked by abundant ions having close by *m*/*z* values. Here, the strongly discriminating imaging software (Δ*m*/*z* = 0.001) coupled with high mass resolution mass spectrometer (maximum 140 000 resolving power) ensures accurate MSI assignment for different metabolites. In **Figure** **5**A, though the three metabolites showed very close *m*/*z* values (red, *m*/*z* 369.0229; green, *m*/*z* 369.0998; blue, *m*/*z* 369.2297), their specific distributions were still detected in rat kidney. The metabolites at *m*/*z* 808.5143 (red) and *m*/*z* 808.5557 (green) demonstrated a complementary spatial distribution in rat brain (Figure 5B).

## 3 Functional Metabolites Based Molecular Histology

In situ profiling the spatial distribution of functional metabolites in tissue is conducive to pathological diagnosis, pharmaceutical mechanisms study, and biomarker discovery at the molecular level. Here, we further validated the wide coverage AFADESI‐MSI method by analyzing highly heterogeneous biological samples, such as rat kidney, rat brain, and human esophageal cancer tissue.

**Figure 6:**
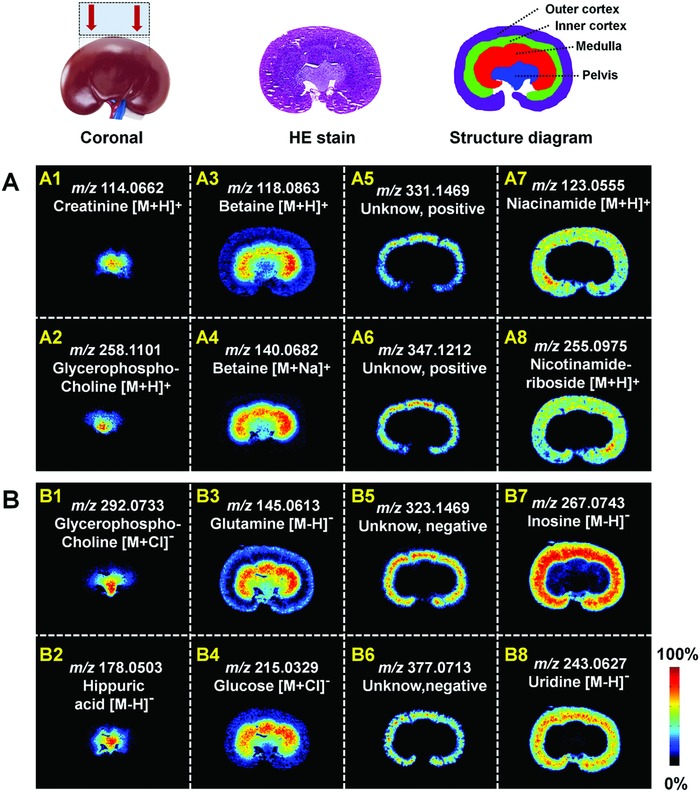
MS images of representative metabolites in rat kidney. A) Positive ion mode. B) Negative ion mode.

Kidney is composed of cortex, medulla, and central pelvis. Its coronal frozen sections were employed to perform AFADESI‐MSI analysis in both positive and negative ion mode for histological examination. **Figure** **6** illustrates the representative tissue‐specific metabolites in rat kidney. It suggests that creatinine (Figure 6A1), a significant indicator of renal function, exclusively distributed in renal pelvis. The ion abundance of the important osmoregulation organic solute glycerophosphocholine (Figure 6A2/6B1) increased sharply from the cortex to medulla and pelvis; its intensity range covered three orders of magnitude (Figure S18, Supporting Information), which was very consistent with previous report.^12^ Hippuric acid (Figure 6B2) as the most frequently used biomarker for occupational exposure to toluene, been linked to antioxidant amino acids and vitamins in recent research,^13^ is mainly located in renal pelvis. Renal medulla manages the reabsorption of amino acids, glucose, water, and salts. As shown in Figure 6, more glutamine (Figure 6B3) and glucose (Figure 6B4) being present in the medulla is beneficial from renal reabsorption. Interestingly, [M+H]^+^ of betaine (*m*/*z* 118.0863, Figure 6A3) just presented a relative stronger ion intensity in the medulla than cortex; however, the [M+Na]^+^ of betaine (*m*/*z* 140.0682, Figure 6A4) almost exclusively appeared in the medulla that was in accordance with the reabsorption capacity for sodium salt in medulla.^14^ In addition, the spatial difference is also likely to be caused by cationization. Niacinamide and nicotinamide riboside gained enough attention for their fundamental roles in the procession of NAD^+^,^15^ and they distributed throughout the whole cortex (Figure 6A7/8). Some metabolite ions, such as *m*/*z* 331.1469 and *m*/*z* 347.1212 in positive ion mode (Figure 6A5/6), *m*/*z* 323.1469 and *m*/*z* 377.0713 in negative ion mode (Figure 6B5/6), displayed an exclusive distribution at inner cortex. There was a sharp decrease in the abundance of nucleosides in the medulla. For example, the ion intensities of inosine (Figure 6B7) and uridine (Figure 6B8) were much stronger in renal cortex than medulla. Conversely, nucleotides which are composed of nucleosides and phosphate, demonstrated stronger ion intensities in renal medulla (Figure S19, Supporting Information). It shows that inosine monophosphate (IMP), adenosine monophosphate (AMP), guanosine monophosphate (GMP), and uridine monophosphate (UMP) almost just located in renal medulla. This may provide a clue to relative active nucleotide cycle in medulla, which was caused by the high ATP‐demanding in the process of reabsorption.

**Figure 7:**
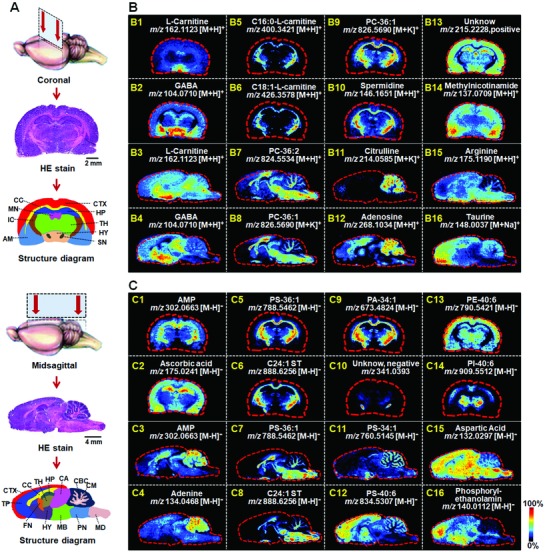
MS images of representative metabolites in rat brain. A) HE stain and structure diagram of rat brain on coronal and midsagittal plane. B) MS images of representative metabolites in positive ion mode. C) MS images of representative metabolites in negative ion mode. AM, amygdala; CA, cerebral aqueduct; CBC, cerebellar cortex; CC, corpus callosum; CM, cerebellar medulla. CTX, cerebral cortex; FN, fornix; HP, hippocampus; HY, hypothalamus; IC, internal capsule; MB, middle brain; MD, medulla; MN, mediodorsal nucleus; PN, pons; SN, substantia nigra; TH, thalamus; TP, telencephalon.

Brain, as the most complex organ in organism, regulates the majority of the body and mind's functions. Thousands of distinguishable areas can be identified within brain based on fine distinctions of neural structure, chemistry, and connectivity. Frozen coronal and midsagittal sections of rat brain were successively subjected to metabolites based histological examination. The distribution of lipids and many small molecule metabolites show perfect correlations with specific anatomical structures of the brain (**Figure** **7**). γ‐Aminobutyric acid (GABA, Figure 7B2/4), an important inhibitory neurotransmitter,^16^ primarily located in the hypothalamus and substantia nigra. In the brain, carnitines alter membrane composition, enhance antioxidant activity, increase mitochondrial function.^17^ Similar to GABA, l‐carnitine (Figure 7B1/3) was primarily found in the hypothalamus. However, the long‐chain acylcarnitines, such as C16:0‐l‐carnitine and C18:1‐l‐carnitine (Figure 7B5/6), almost exclusively appeared in the corpus callosum and internal capsule. Adenosine, an important neuromodulator,^18^ was scarce in the corpus callosum, hypothalamus, and thalamus, while it presented high levels in the telencephalon and cerebellum (Figure 7B12). For adenine and adenosine monophosphate, which are closely related to adenosine in metabolic pathway demonstrated a consistent spatial distribution with adenosine (Figure 7C1/3/4). Lipids, especially for phospholipids (PLs), showed specific spatial distribution in brain. For example, phosphatidylcholine (PC)‐36:1 (Figure 7B9), phosphatidylserine (PS)‐36:1 (Figure 7C5), C24:1‐ST (Figure 7C6), and phosphatidic acid (PA)‐34:1 (Figure 7C9) almost exclusively appeared in the corpus callosum and internal capsule on the coronal section, while phosphatidylethanolamine (PE)‐40:6 (Figure 7C13) showed a totally opposite spatial distribution. Phosphatidylinositol (PI)‐40:6 mainly distributed in corpus callosum and thalamus (Figure 7C14). Interestingly, the distributions of some lipids are complementary across the midsagittal plane of brain. For instance, PS‐36:1 (Figure 7C7) and C24:1‐ST (Figure 7C8) indicate the cerebellum medulla, while PS‐34:1 (Figure 7C11) and PS‐40:6 (Figure 7C12) represent the cerebellum cortex. In addition to the above mentioned metabolites, region‐specific ascorbic acid, spermidine, arginine, citrulline, taurine, etc., are also illustrated in Figure 7.

**Figure 8:**
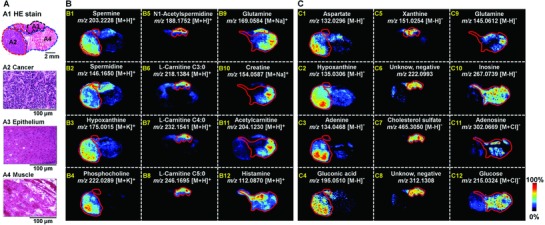
MS images of representative metabolites in human esophageal cancer tissue. A) HE stain of esophageal cancer tissue. B) MS images in positive ion mode. C) MS images in negative ion mode.

Metabolic reprogramming is critical to oncogenesis, and a global in situ metabolic profiling facilitates understanding of the profound reorganization. Esophageal cancer is one of the most deadly diseases. Its cancer cell successively invade esophageal wall from mucosa, submucosa, and muscularis in the progression of cancer.^19^ Here, an esophageal cancer tissue sample was collected by surgical resection, having previously approved by the local Ethical Review Board. Cryosection of esophageal cancer tissue was analyzed by AFADESI‐MSI to perform histological examination for discovering dysregulated functional metabolites to understand the complex molecular mechanism in cancer progress. MS images of typical specific metabolites in cancer tissue and paracancerous tissue are illustrated in **Figure** **8**. Polyamines, including spermine and spermidine, control gene expression at the transcriptional and translational levels. Furthermore, polyamine catabolism enzymes change rapidly during tumorigenesis, and were regarded as potential targets for chemotherapy and chemoprevention.^20^ Our MSI result suggests that spermine (Figure 8B1) and spermidine (Figure 8B2) were upregulated in cancer tissue, corresponding to the stronger cell proliferative ability. Hypoxanthine and adenine are two important nitrogen base, mainly distributed in cancer tissue (Figure 8B3/C2/C3), while their respective nucleoside inosine and adenosine were downregulated in cancer (Figure 8C10/11), and this may help to understand the highly expressed purine nucleoside phosphorylase in cancer tissue at the metabolite level.^21^ Glutamine is a critical metabolite that participates in energy metabolism, redox homeostasis, nucleotide, and amino acid biosynthesis.^22^ Moreover, as a major tricarboxylic acid (TCA) cycle carbon source in cancerous cells, glutamine is necessary to support cancerous cells growth and proliferation.^23^ The frequently activate glutamine consumption in cancerous cells was further proved by our MSI assay. As illustrated in Figure 8B9/C9, the ion intensity of glutamine was weaker in cancer tissue than paracancerous tissue. The high demand of cancerous cells for glucose to produce ATP, support bioenergetic and macromolecular synthesis has long been recognized.^24^ MS imaging result indicates that glucose was significant downregulated in cancer tissue (Figure 8C12), which was consistent with increased glucose consumption in cancerous cells. Moreover, it is interesting to note that some metabolites demonstrated epithelium‐specific distribution. For example, N1‐acetylspermidine (Figure 8B5), l‐carnitine C3:0 (Figure 8B6), l‐carnitine C4:0 (Figure 8B7), l‐carnitine C5:0 (Figure 8B8), cholesterol sulfate (Figure 8C7), etc., mainly distributed in paracancerous epithelium. Lipids, especially those with high ionization efficiency, can be observed easily in AFADESI‐MSI. For example, the MS image intensities of representative fatty acid (FA), lysophosphatide (Lyso‐PL), PC, PE, phosphatidylglycerol (PG), and phosphatidylinositol (PI) were stronger in cancer tissue than paracancerous epithelium and muscle; high relative abundances of sphingomyelin (SM) species were observed in muscular tissue, while PS presented an epithelium‐specific distribution (Figure S22, Supporting Information).

## 4 Discussion

Since the spray solvent play an important role in the course of extracting and ionizing molecules from tissue surface,^25^ it is necessary to optimize spray solvent for metabolites' high sensitive and wide coverage detection. In this contribution, a series of uniform and repeatable tissue models were prepared and then analyzed in different spray solvent systems. Ion intensities and MS images of representative endogenous metabolites at different *m*/*z* ranges (*m*/*z* 70–150, *m*/*z* 150–250, *m*/*z* 250–600, *m*/*z* 600–1000) in different solvent systems were evaluated (Figures S5–S7, Supporting Information). Finally, solvent system ACN/H_2_O (8:2) was chosen for its ability to dissolve and transport more metabolites.

Lipids, as important molecules in cellular processes, have received more attention in previous reported ambient MSI based histological research because of their high ionization efficiency. However, the other small molecule metabolites, such as amino acids, nucleosides, carbohydrates, etc., which possess extensive biofunction are not getting enough attention. In this study, a remarkable enhancement of sensitivity was achieved by introducing high‐rate air flow to AFADESI ion source and optimizing spray solvent system for different metabolites. Maximum ion intensities exceeded 1.0E^6^ counts in both positive and negative ion mode (Figure S2, Supporting Information). The improved sensitivity contributes to broaden metabolites coverage and map the spatial distribution of low‐abundance small molecule metabolites in tissue.

So far, tens of thousands of endogenous metabolites were reported in organism. Moreover, a high proportion of them have very similar exact mass values. The metabolites may present an entirely different spatial distribution at different microregion for diverse biological functions. Therefore, high mass resolution mass spectrometer and highly discriminatory imaging software are essential to visualize spatial distribution of different metabolites, especially for those *m*/*z*‐closed metabolites. Here, dedicated imaging software was developed based on the C++ programming language, and the minimum bin width was 0.001 mass units. The strong discriminability ensures precise image assignment for *m*/*z*‐closed metabolites in histological examination. However, it is still challenging to assign different metabolites with identical *m*/*z* values, especially in complex biological samples. Notably, tandem MS imaging is an underused technique, which is capable of imaging metabolites with greater molecular specificity than based on ion mass alone.[[qv: 3b]] Nevertheless, it is more suitable for targeted analysis of specific metabolites.

Microscopical evaluation of tissue samples which were dyed using distinct histochemical staining method is still the primary technique for histological and pathological diagnosis. Yet, the staining techniques were always performed in a targeted manner for limited known molecules at the same time, and the time‐consuming procedure made it difficult for rapid pathological diagnosis. While, the AFADESI‐MSI based histological examination method can provide thousands of metabolites spatial information in 40 min for a 1 cm × 1 cm tissue section, including ≈15 min for tissue section preparation and ≈25 min for metabolites imaging. There is no doubt that this rapid imaging method would facilitate histological and pathological diagnosis and accelerate intraoperative decision‐making.

## 5 Conclusion

In this study, a sensitive and wide coverage AFADESI‐MSI method was developed to in situ analyze global metabolites on tissue and discover tissue‐specific biomarkers. More than 1500 metabolites including cholines, polyamines, amino acids, carnitines, nucleosides, nucleotides, nitrogen bases, organic acids, carbohydrates, cholesterol sulfate, cholic acid, lipids, and other small molecule metabolites were detected. It is important to note that thousands of structure‐specific and functionally relevant metabolites' images can be acquired on tissue from an untargeted AFADESI‐MSI analysis. This is a major advantage over histochemical staining or targeted immunohistochemical analysis. Moreover, it can not only automatically identify and quantify spectral features of anatomically relevant metabolites but also give insights into pathological mechanisms at the molecular level, uncovering the associations between metabolites specific distribution and structural and functional characteristics. This method shows promising applications in tissue‐specific biomarker discovery, in situ metabolomics study, and molecular histological analysis for its wide coverage, high sensitivity, wide dynamic range, rapid analysis procedure, and high chemical specific.

## 6 Experimental Section

The animal experiments were approved by the Animal Care & Welfare Committee, Institute of Materia Medica, Chinese Academy of Medical Sciences and Peking Union Medical College (Beijing, China). Rat brain, kidney, and liver tissue were acquired after sacrificed by anesthesia. Human esophageal cancer sample was collected by surgical resection, having previously approved by the local Ethical Review Board of Linzhou Esophageal Cancer Hospital (Linzhou, China) and the patients provided written informed consent. For additional details see the Supporting Information. Extracted adducted ions were compared with free databases using exact molecular weights, combining the isotope abundance from HR‐MS help to give the possible elemental composition.^26^ Then, representative metabolites were performed high‐resolution MS/MS directly from tissue sections.

## Conflict of Interest

The authors declare no conflict of interest.

## Supporting information

Supplementary
