# Supplementary material for: A Sensitive and Wide Coverage Ambient Mass Spectrometry Imaging Method for Functional Metabolites Based Molecular Histology
Source: Adv Sci (Weinh). 2018 Oct 7;5(11):1800250. doi: 10.1002/advs.201800250 (PMC6247026; doi:10.1002/advs.201800250)
Supplement: Supplementary file 1 — Supplementary [file ADVS-5-1800250-s001.pdf]

Copyright WILEY-VCH Verlag GmbH & Co. KGaA, 69469 Weinheim, Germany, 2018.

ADVANCED SCIENCE
Open Access

Supporting Information

for *Adv. Sci.,* DOI: 10.1002/advs.201800250

A Sensitive and Wide Coverage Ambient Mass Spectrometry Imaging Method for Functional Metabolites Based Molecular Histology

*Jiuming He, Chenglong Sun, Tiegang Li, Zhigang Luo, Luojiao Huang, Xiaowei Song, Xin Li, and Zeper Abliz**

Copyright WILEY-VCH Verlag GmbH & Co. KGaA, 69469 Weinheim, Germany, 2016.

Supporting Information

A Sensitive and Wide Coverage Ambient Mass Spectrometry Imaging Method for Functional Metabolites Based Molecular Histology

*Jiuming He, Chenglong Sun, Tiegang Li, Zhigang Luo, Luojiao Huang, Xiaowei Song, Xin Li, and Zeper Abliz**

Solvents and Reagents

HPLC-grade organic solvents, including acetonitrile, methanol, isopropanol and berberine chloride were purchased from Merck (Muskegon, MI). Purified water was obtained from Wahaha (Hangzhou, China). The Wistar rats were purchased from Vital River Laboratory Animal Technology Co (Beijing, China). Saline was obtained from Cisen Pharmaceutical Co. (Jining, China). D_9-choline chloride was purchased from Cambridge Isotope Laboratories, Inc.

AFADESI-MSI platform

The original ion source of Q-Orbitrap mass spectrometer (Q Exactive, Thermo Scientific Bremen, Germany) was removed, a home-built AFADESI ion source was installed on the benchtop, 3D translational stage controlled by custom-developed software was placed under the ion source. Schematic of AFADESI-MSI platform based on hybrid Q-Orbitrap mass spectrometer was shown in Figure S1. SC100 series stepper motor (Beijing Optical Century Instrument Co., Beijng, China) was used to control the 3D mobile platform.

Tissue homogenate model preparation

For AFADESI-MSI, uniform and repeatable tissue sample was crucial for optimization of analytical conditions. A PVC adhesive sticker with 2*5 mm quasi-rectangular hole was posted on microscope slide. Then, 3 μL liver homogenate was add to the quasi-rectangular hole by micro-pipette. Dried in vacuum after 15 min, the homogenate model was formed. The detailed process to prepare tissue homogenate model were given in Figure S3.

Sample preparation and process

Rat brain, kidney, liver tissue were acquired after sacrificed by anesthesia. Human esophageal cancer sample was collected by surgical resection, having previously approved by the local Ethical Review Board. Then the collected bio-samples were cut into 12 μm sections at -20°C

on cryostat microtome (CM 1860 UV, Leica Microsystems, Wetzlar, Germany) and thaw-mounted onto microscope slide. One set of adjacent tissue sections were fixed in acetone and then were dyed with H&E for histological observation. After dried in vacuum for 15 min, the cryosections were employed to perform the (±) MSI analysis in Full MS (70-1000 *m/z*) scan mode.

MSI data acquisition

The solvent flow was set at 5 μL/min for ex vivo biosamples. The MSI experiments were performed by continuously scanning the tissue surface in the *x*-direction at a constant rate of 200 μm/sec, separated by a 200 μm vertical step in *y*-direction. The sprayer and transport tube voltages were set at 7000 V and 3000 V, respectively. The extracting gas flow was 45 L/min, and the capillary temperature was 350°C. The endogenous metabolites were monitored using Full MS scan mode (*m/z* range: 70-1000, Automatic Gain Control: 3E6, Maximum injection time 200 ms).

Data processing and analysis

Raw data acquired by Xcalibur 2.3 (Thermo Fisher Scientific Inc.) were converted into .cdf format for image reconstruction. The custom-developed software MassImager^{TM} (in corporation with Chemmind Technologies Co., Ltd) was used for ion image reconstructions, background subtraction and the generation of an average mass spectrum in a region of the interests. The separated sample dataset matrixes were then imported into the Markerview^{TM} software 1.2.1 (AB SCIEX) for peak picking, peak alignment and isotope removing (process spectra options: the mass tolerance was 0.01 Da and the minimum required response was 50). Next, the list was exported in .txt format for later statistical analysis.

Optimization of spray solvent system

ACN/H_2O (5:5), ACN/H_2O (8:2), ACN/IPA/H_2O (4:4:2), ACN/IPA/H_2O (6:2:2), MeOH/H_2O (5:5), MeOH/H_2O (8:2), MeOH/IPA/H_2O (4:4:2) and MeOH/IPA/H_2O (6:2:2) were successively tried to seek the optimal spray solvent system for different metabolites. Intensities of representative endogenous metabolites at different *m/z* ranges (*m/z* 70-150, *m/z* 150-250, *m/z* 250-600, *m/z* 600-1000) in different solvent systems were evaluated. The precision of tissue homogenate model was illustrated in Figure S4. Relative numbers of endogenous metabolites in different spray solvent systems were shown in Figure S5. Ion intensities of all the detected metabolites were firstly assessed using heat map (Figure S6). Then, the ion intensities of representative endogenous metabolites in different solvent systems

were shown in Figure S7. Solvent system ACN/H_2O (8:2) was finally chosen for the prosperity of most metabolites. Under the optimized solvent system, rat brain, rat kidney and human esophageal cancer sample were successively evaluated by AFADESI-MSI. After deducting background ions by MassImager, peak picking, peak alignment and isotope removing by Markerview, more than 1,500 metabolites (when ion intensity approximate 1000 counts in MassImager^{TM}, metabolites have clear MS images, Figure S10) were visualized.

Dynamic range evaluation

Calibration standards samples were prepared by spiking blank liver homogenate with proper volume of standard working solutions of berberine chloride and D_9-choline chloride. For berberine chloride, 0.06, 0.18, 0.54, 1.62, 4.86, 14.6, 43.8 and 87.5 ng/mm^2 tissue homogenate models were prepared. For D_9-choline chloride, 0.4, 1.2, 3.6, 10.8, 32.4, 97.3, 292 and 875 ng/mm^2 tissue homogenate models were prepared. The prepared tissue homogenate models were performed AFADESI-MSI analysis under the optimized solvent system. The specific results were illustrated in Figure S17. It suggests that the calibration curves were linear over the concentration range of 0.06-87.5 ng/mm^2 for berberine and 0.4-875 ng/mm^2 for D_9-choline, with a correlation coefficient $r \geq 0.99$ for the two analytes.

Analyte identification

Extracted adducted ions at different *m/z* ranges were compared with free databases HMDB (http://hmdb.ca/), Metlin (http://metlin.scripps.edu), and LIPID MAPS (http://www.lipidmaps.org/) using exact molecular weights and a mass accuracy of less than 5 ppm, combining the isotope abundance from HR-MS help to give the elemental composition and possible list of endogenous metabolites. Subsequently, representative metabolites of different kinds at different *m/z* range were performed high resolution MS/MS directly from tissue sections. The flow rate of spray solvent was 5 μL/min, the scan rate was set at a constant rate of 200 μm/sec in the tissue surface. The ions of interest were listed as the targets, with the NCE value set at 25%, 35%, and 45% in targeted-MS2 scan mode. The resolving power was set at 17500 for MS/MS acquisition with the AGC value at 3E6 and maximum injection time at 200 ms. The structure-specific pattern ions of the target analyte were used for further identification. The results were listed in the Table S1,S2 and Figure S24- S52.

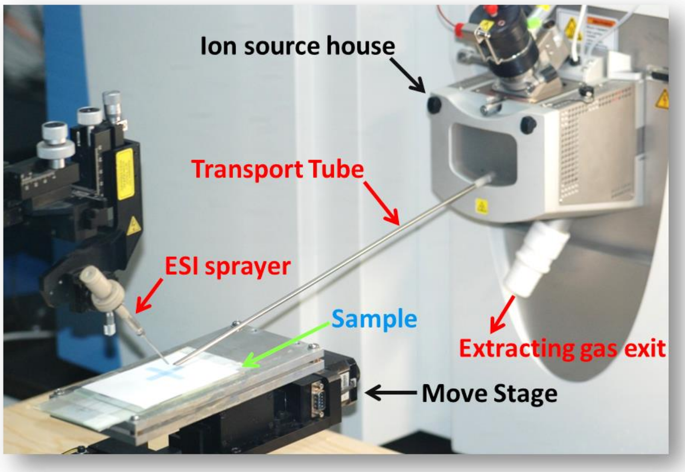

Figure S1. AFADESI-MSI platform based on hybrid Q-Orbitrap mass spectrometer.

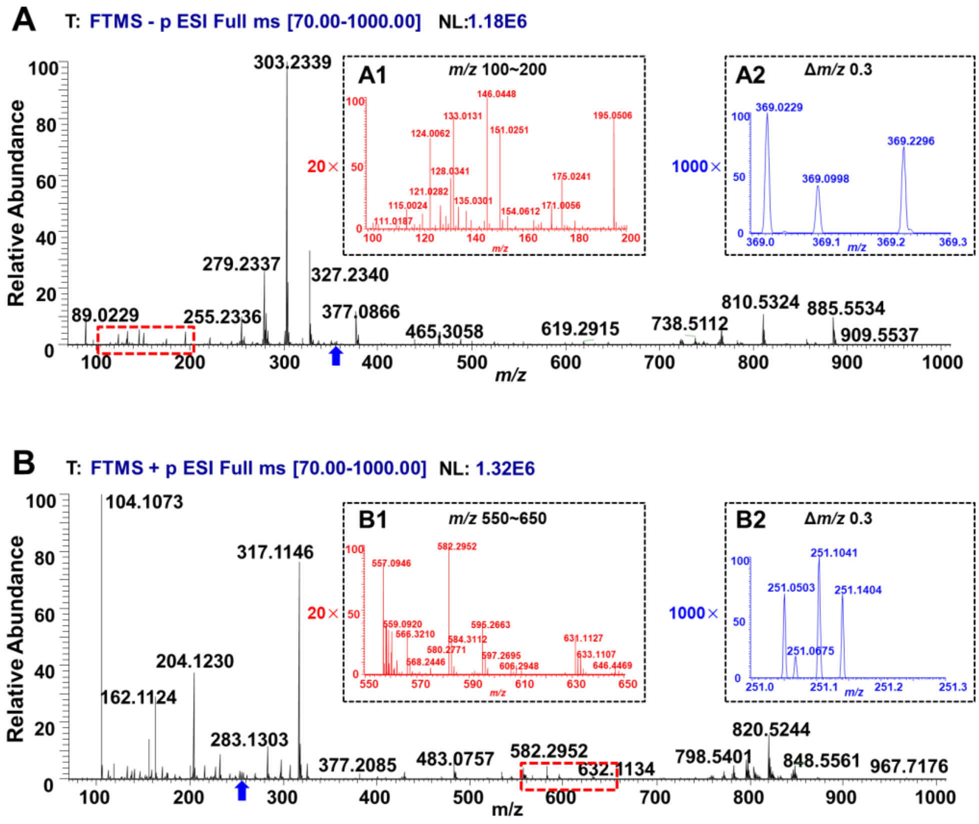

Figure S2. Representative AFADESI-MS spectra in positive and negative ion mode. Representative AFADESI-MS spectra in positive and negative ion mode. (A) Negative AFADESI-MS spectra. (A1) 20 Fold magnification at *m/z* 100~200. (A2) 1000 Fold magnification at *m/z* 369 within 0.3 bin width. (B) Positive AFADESI-MS spectra. (B1) 20 Fold magnification at *m/z* 550~650. (B2) 1000 Fold magnification at *m/z* 251 within 0.3 bin width.

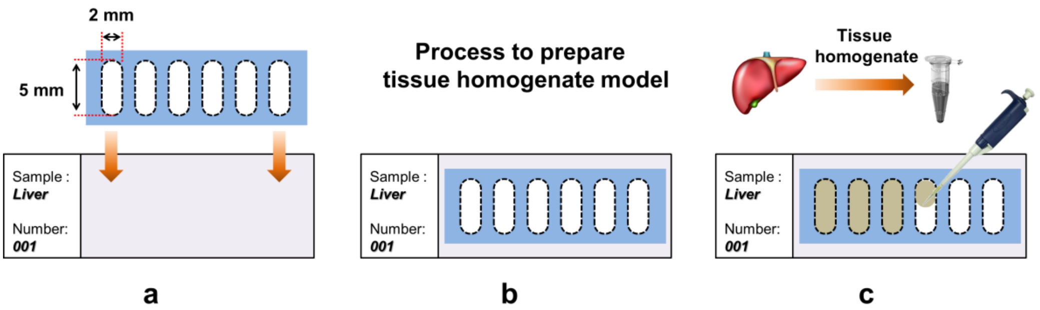

Figure S3. The process to prepare tissue homogenate model. (a) A PVC adhesive sticker with 2*5 mm quasi-rectangular hole was prepared. (b) Adhesive sticker was posted on microscope slide. (c) 3 µL liver homogenate was add to the quasi-rectangular hole by micro-pipette.

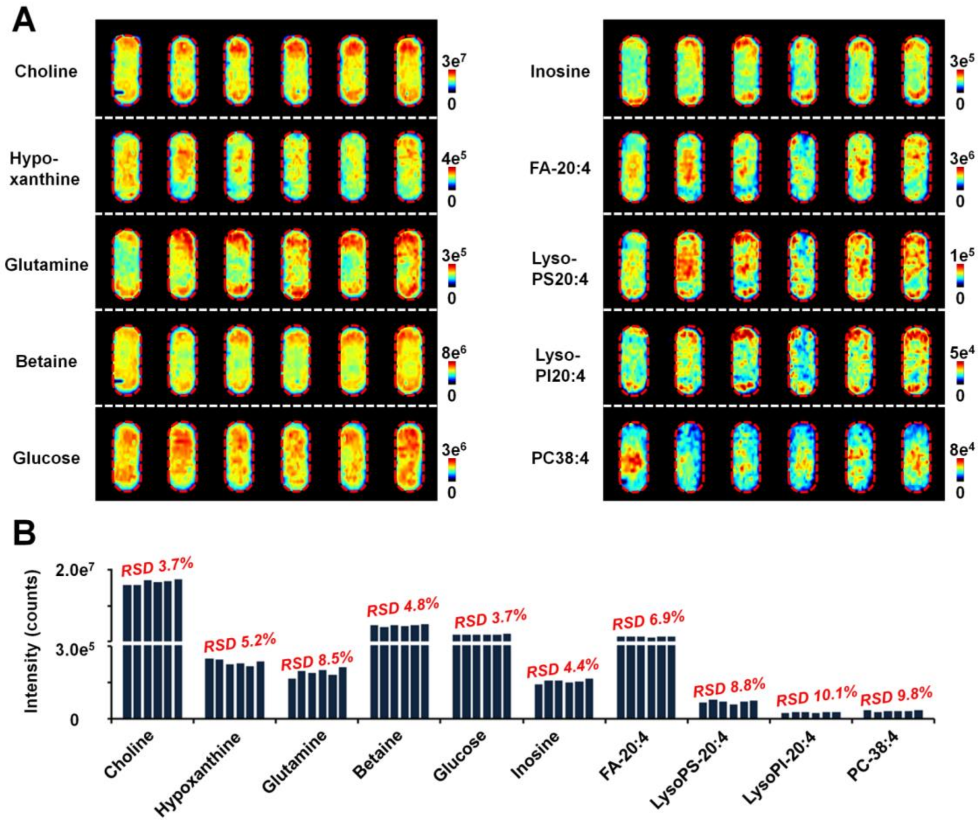

Figure S4. The precision of tissue homogenate model. (A) MS images of representative metabolites at different *m/z* ranges. (B) Statistic analysis of representative metabolites at different m/z ranges.

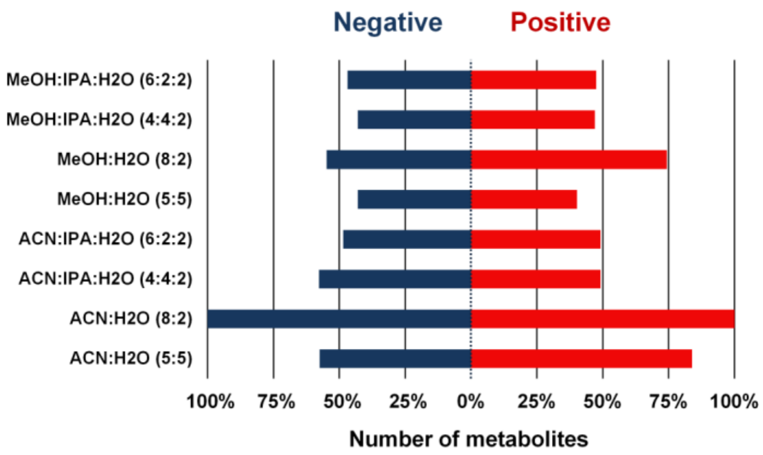

Figure S5. Relative number of endogenous metabolites in different spray solvent systems.

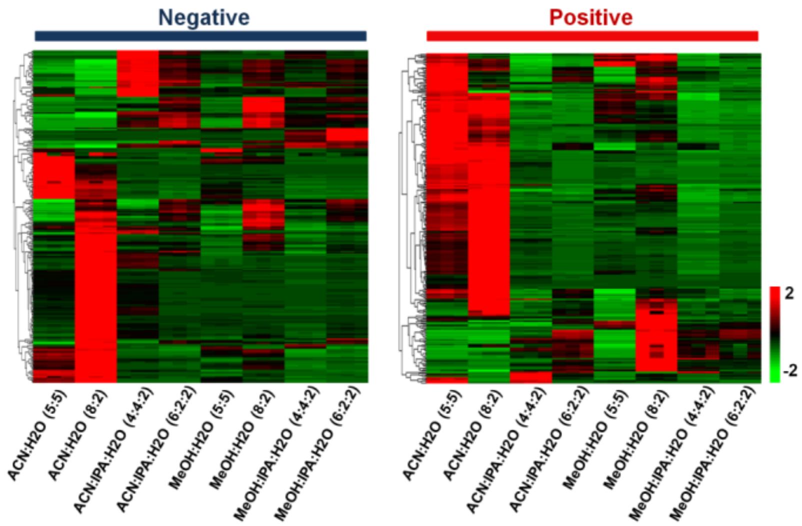

Figure S6. Heat map of metabolite relative ion intensity in different spray solvent systems.

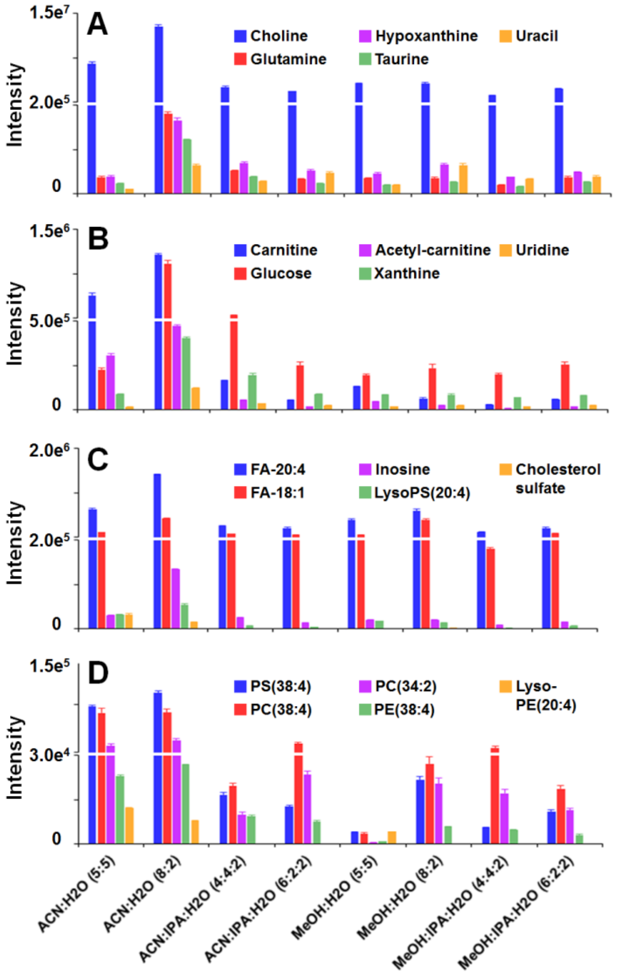

Figure S7. The ion intensity of representative endogenous metabolites in different solvent systems. (A) The ion intensities of choline,hypoxanthine, uracil, glutamine and taurine at *m/z* 70-150 range in different solvent systems. (B) The ion intensities of carnitine,acetyl-carnitine, uridine, glucose and xanthine at *m/z* 150-250 range in different solvent systems. (C) The ion intensities of fatty acid (FA)-20:4, inosine, cholesterol sulfate, FA-18:1 and lysophosphatidylserine (LysoPS)-20:4 at *m/z* 250-600 range in different solvent systems. (D) The ion intensities of phosphatidylserine (PS)-38:4, phosphatidylcholine (PC)-34:2, lyso phosphatidylethanolamine (LysoPE)-20:4, PC-38:4 and PE-38:4 at *m/z* 600-1000 range in different solvent systems.

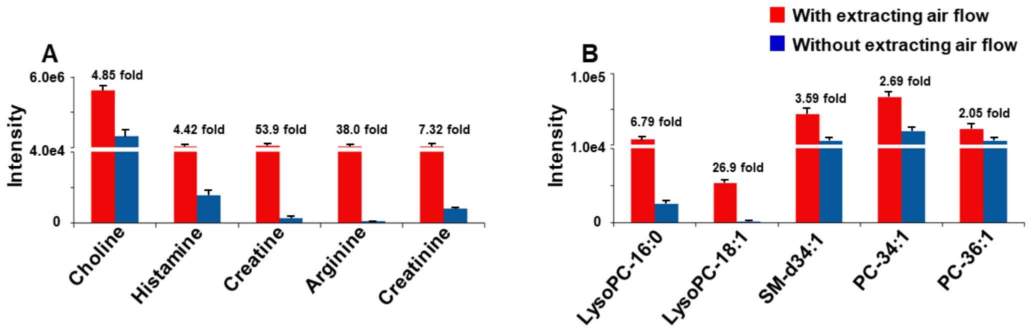

Figure S8. The ion intensity of representative endogenous metabolites with or without extracting air flow (A, small molecule metabolites. B, lipids).

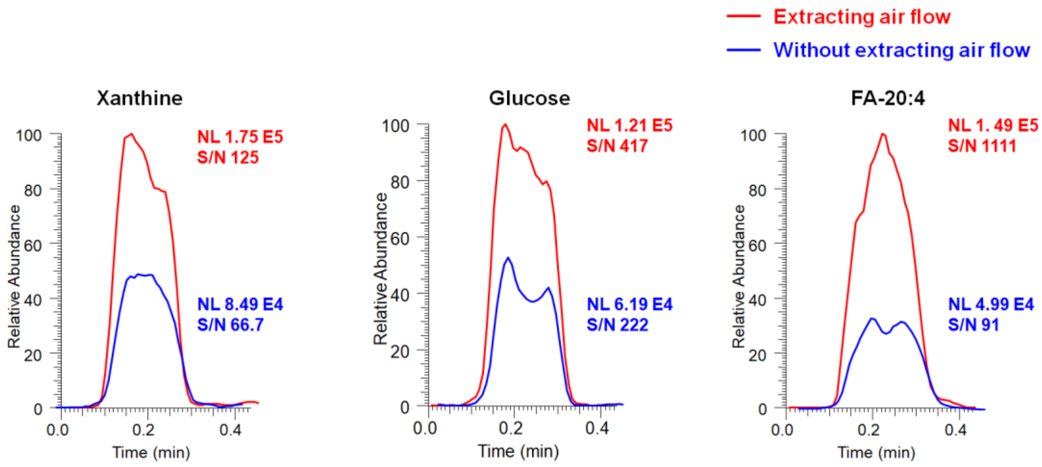

Figure S9. The ion intensities and S/N (signal/noise) values of representative endogenous metabolites with or without extracting air flow.

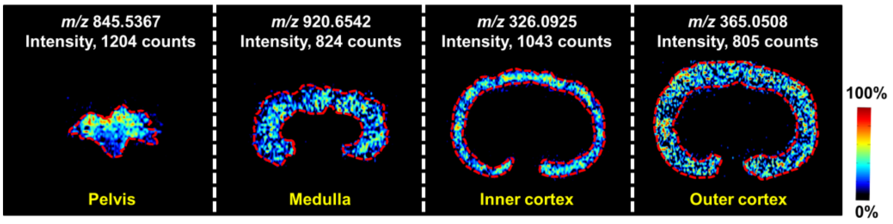

Figure S10. MS images of metabolites with intensity about 1000 counts in MassImager^{TM}.(When ion intensity>1000 counts in MassImager^{TM}, metabolites have clear MS images)

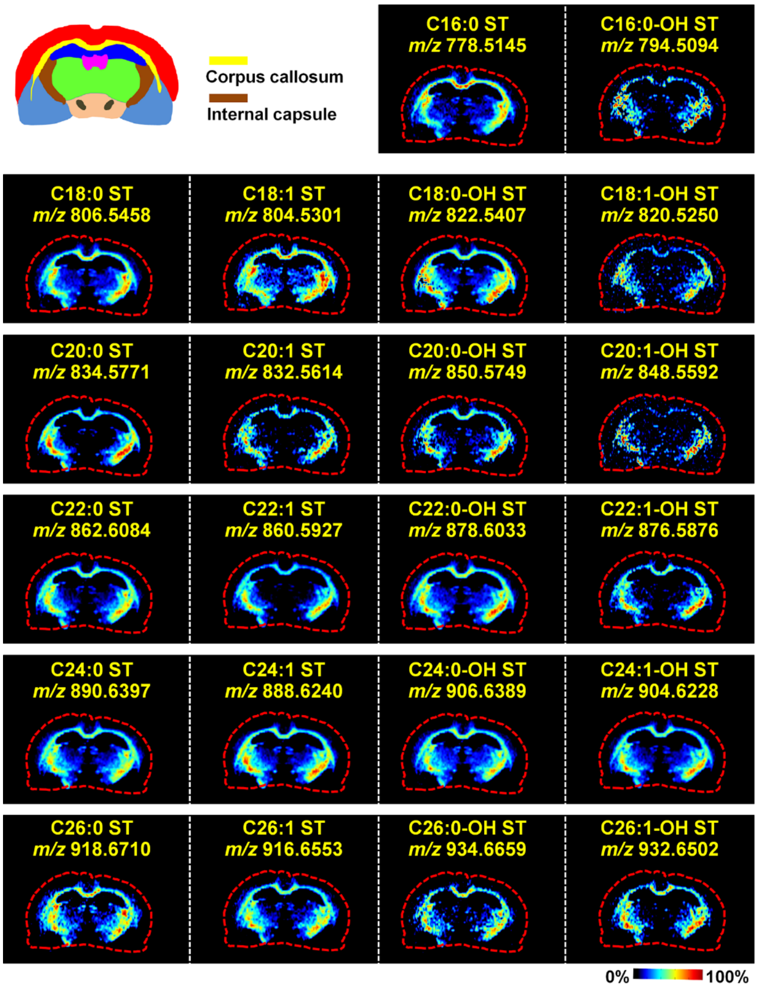

Figure S11. MS images of representative sulfatide (ST) in rat brain.

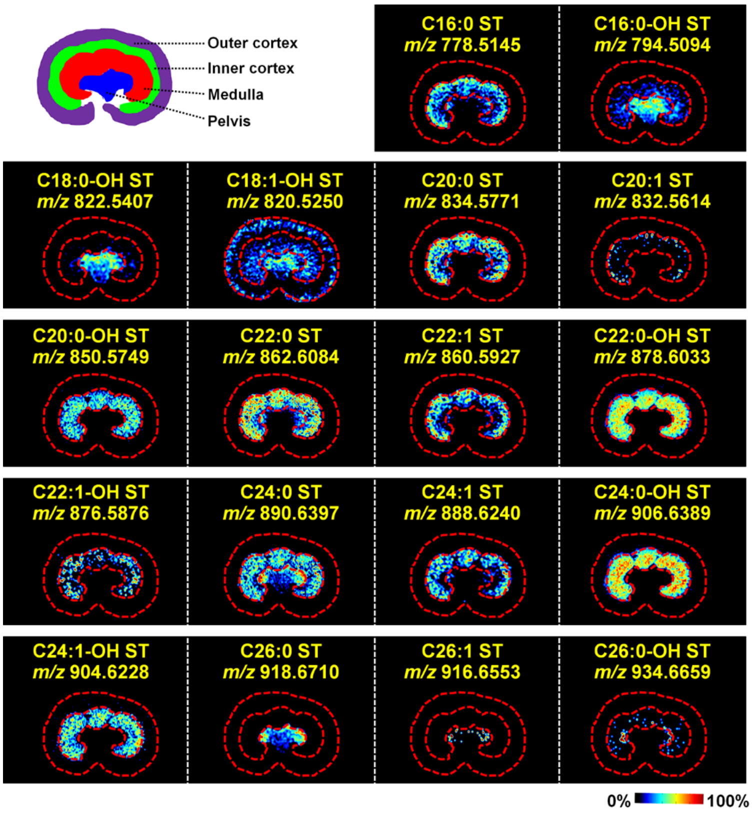

Figure S12. MS images of representative sulfatide (ST) in rat kidney.

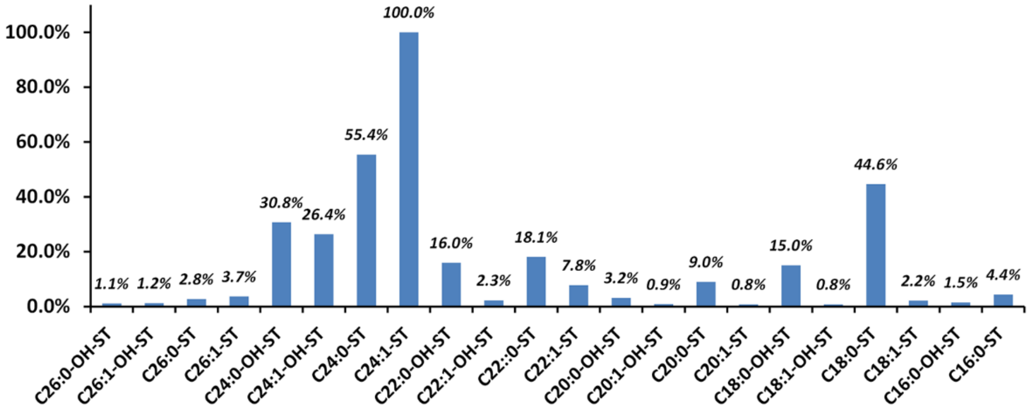

Figure S13. The relative content of different sulfatide (ST) in rat brain.

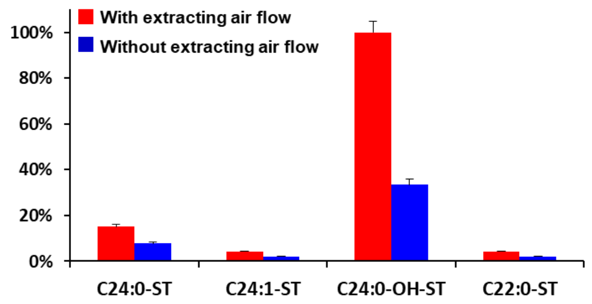

Figure S14. Relative ion intensities of representative sulfatides (ST) with or without extracting air flow

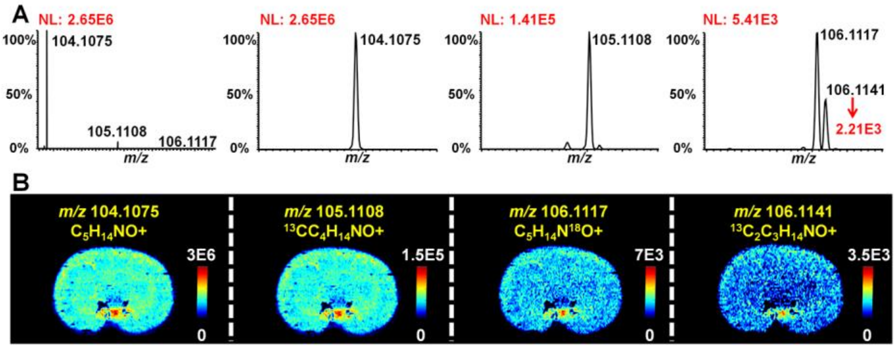

Figure S15. MS spectra and MS images of choline and its isotope ions in rat kidney. (A) MS spectra of choline and its isotopic ions in rat kidney. (B) MS images of choline and its isotopic ions in rat kidney.

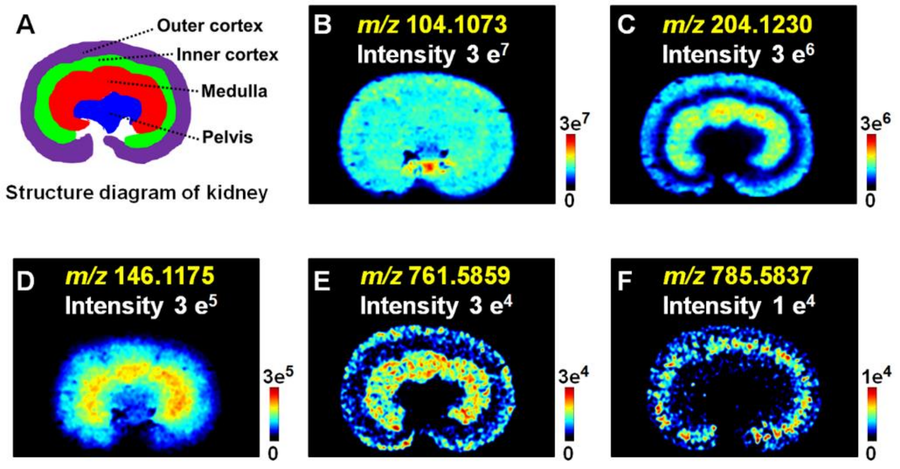

Figure S16. Content differences of representative endogenous metabolites in in kidney tissue. (A) Structure diagram of rat kidney, (B~F) MS images of *m/z* 104.1073, *m/z* 204.1230, *m/z* 146.1175, *m/z* 761.5859, and *m/z* 785.5837.

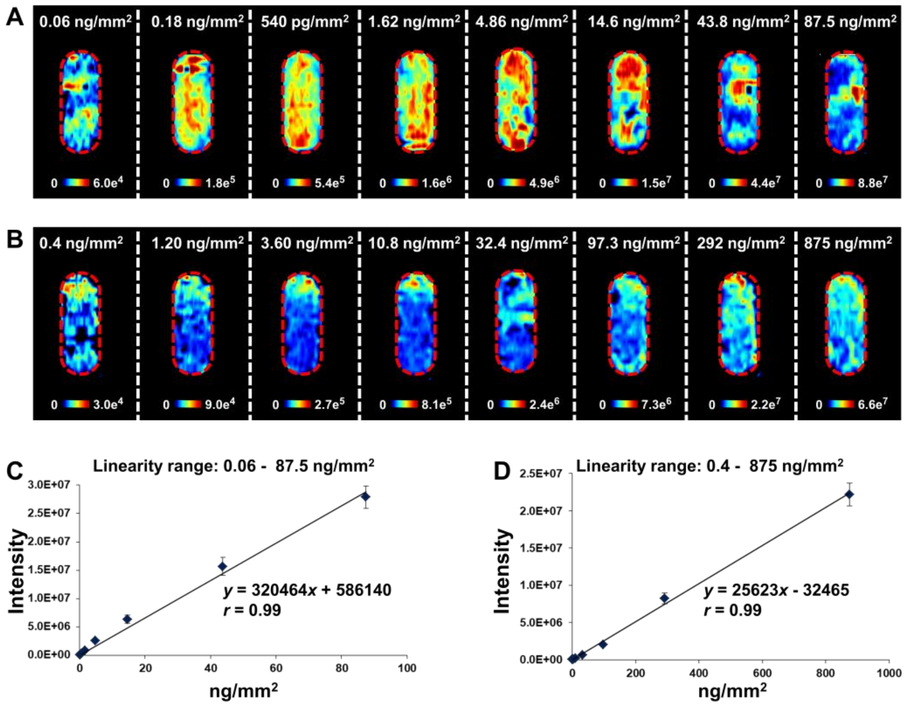

Figure S17. Dynamic range analysis of the high sensitive AFADESI-MSI method. (A) MSI of berberine at different concentration in tissue homogenate models. (B) MSI of D_9-choline at different concentration in tissue homogenate models. (C) Calibration curve of berberine in the linearty of 0.06 – 87.5 ng/mm^2. (D) Calibration curve of D_9-choline in the linearty of 0.6 – 875 ng/mm^2.

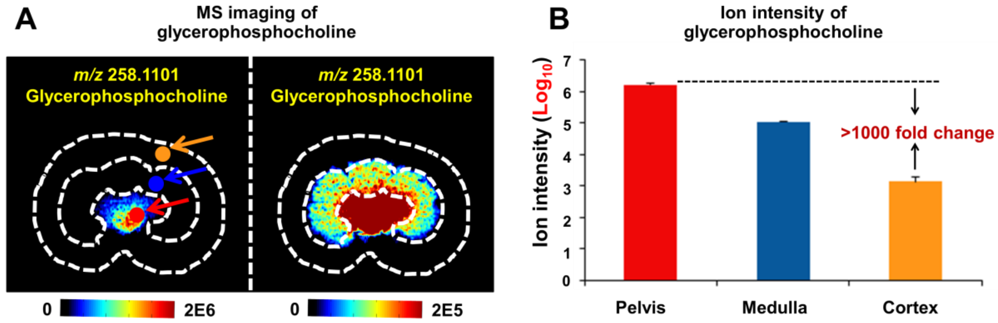

Figure S18. MS image and ion intensity of glycerophosphocholine in rat kidney. (A) MS images of glycerophosphocholine in rat kidney with different intensity threshold. (B) Ion intensity of glycerophosphocholine in different regions of rat kidney.

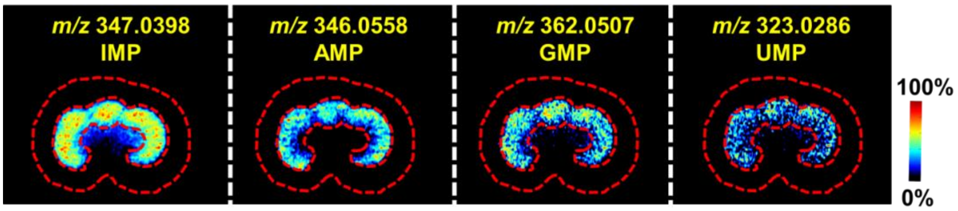

Figure S19. MS images of representative nucleotides in rat kidney.

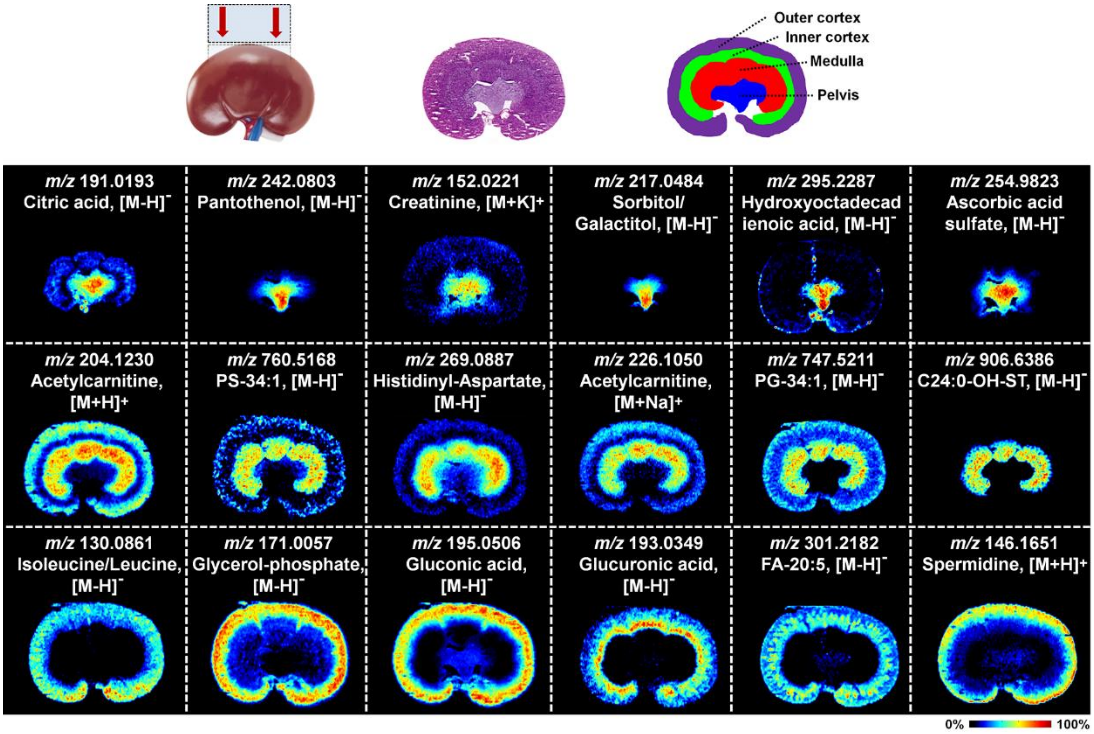

Figure S20. MS images of representative metabolites in rat kidney.

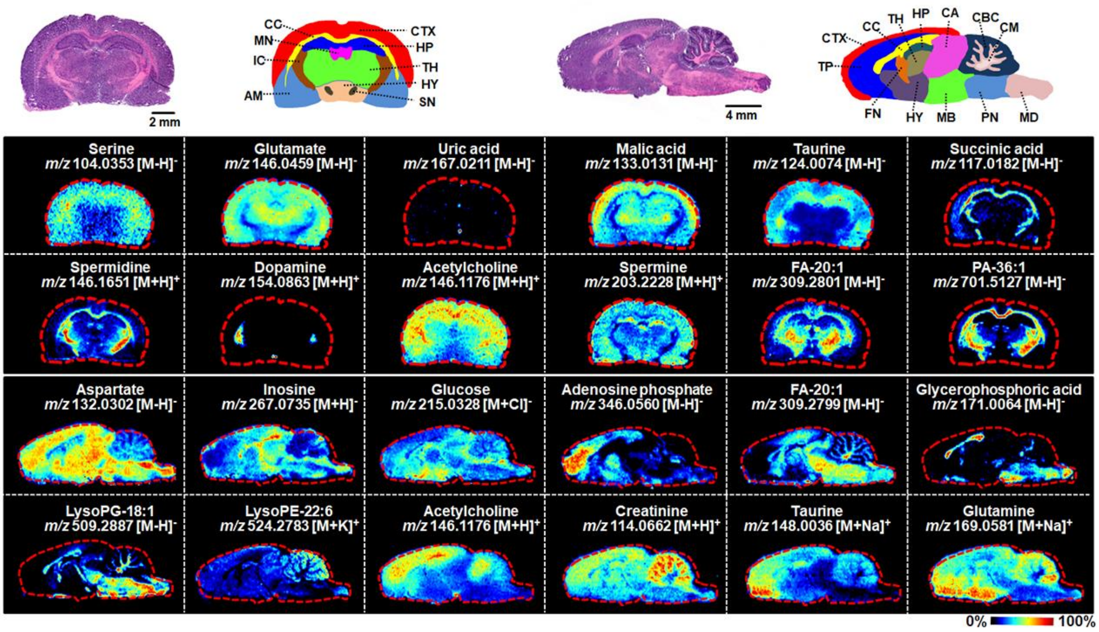

Figure S21. MS images of representative metabolites in rat brain. AM, amygdala; CA, cerebral aqueduct; CBC, cerebellar cortex; CC, corpus callosum; CM, cerebellar medulla. CTX, cerebral cortex; FN, fornix; HP, hippocampus; HY, hypothalamus; IC, internal capsule; MB, middle brain; MD, medulla; MN, mediodorsal nucleus; PN, pons; SN,substantia nigra; TH, thalamus; TP, telencephalon.

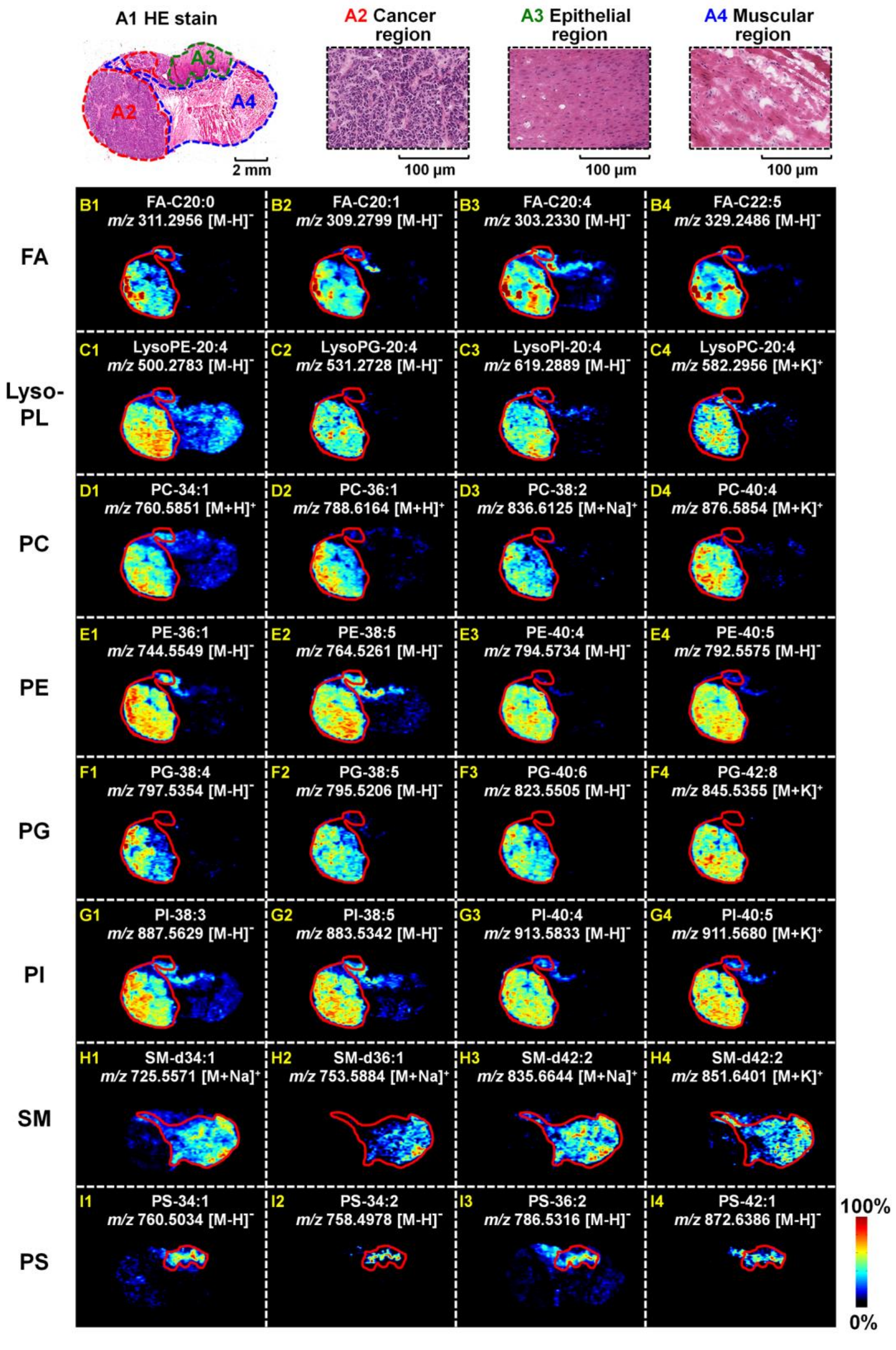

Figure S22. MS images of representative metabolites in human esophageal cancer tissue (FA, fatty acid; Lyso-PL, Lysophosphatide; PC, phosphatidylcholine; PE, phosphatidylethanolamine; PG, phosphatidylglycerol; PI, phosphatidylinositol; SM, sphingomyelin; PS, phosphatidylserine).

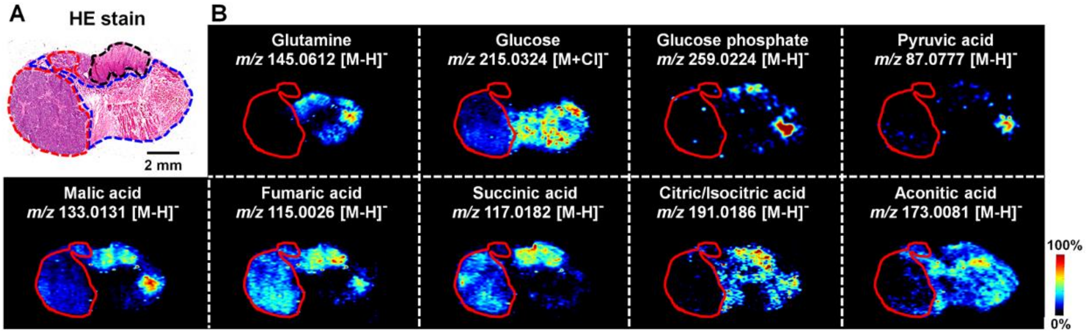

Figure S23. MS images of representative metabolites of TCA (tricarboxylic acid) cycle in human esophageal cancer tissue. A, HE stain of esophageal cancer tissue, red, black, and blue dotted line represent cancer, epithelial, and muscular region, respectively. B, MS images of representative metabolites of TCA cycle.

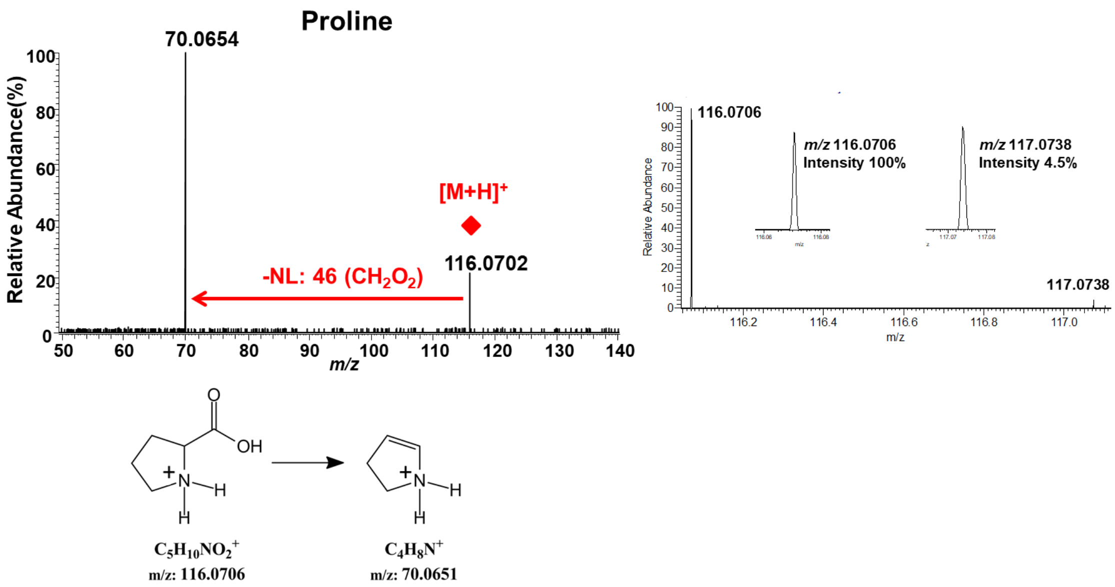

Figure S24. The isotope abundance, MS/MS spectrum and the fragmentation pathway of proline.

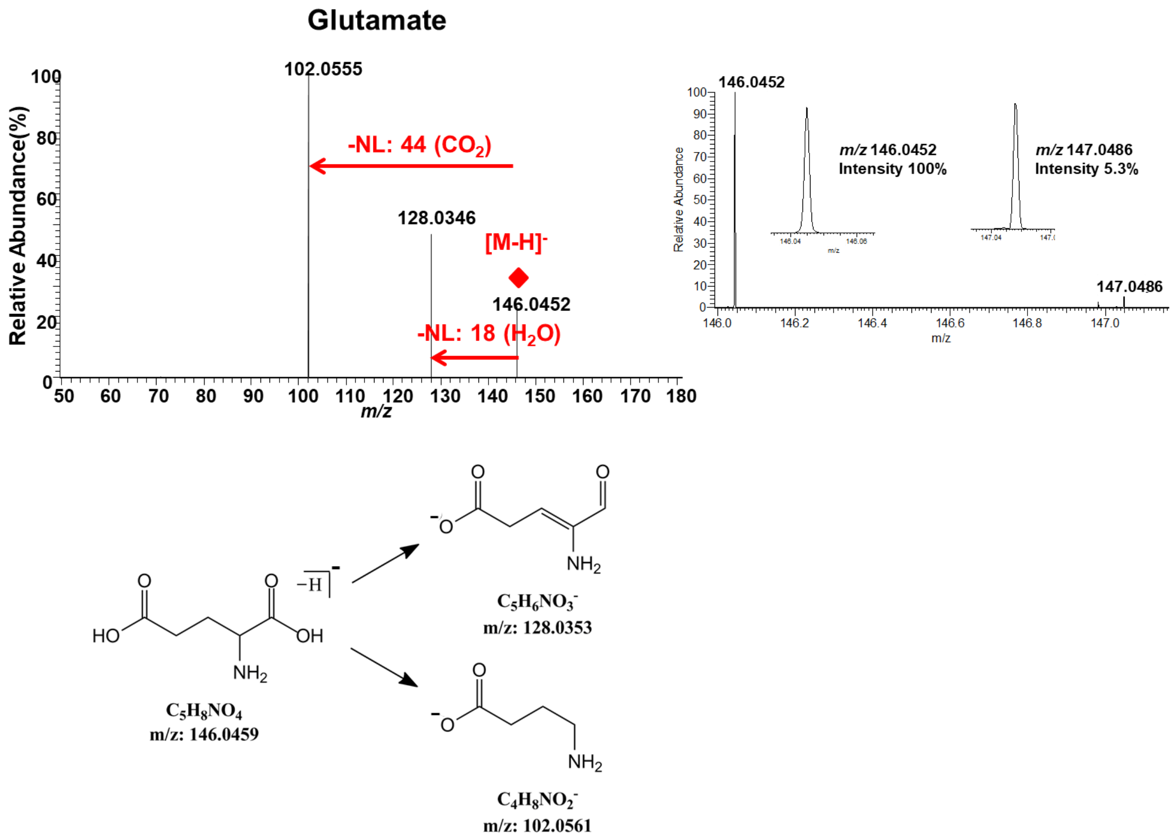

Figure S25. The isotope abundance, MS/MS spectrum and the fragmentation pathway of glutamate.

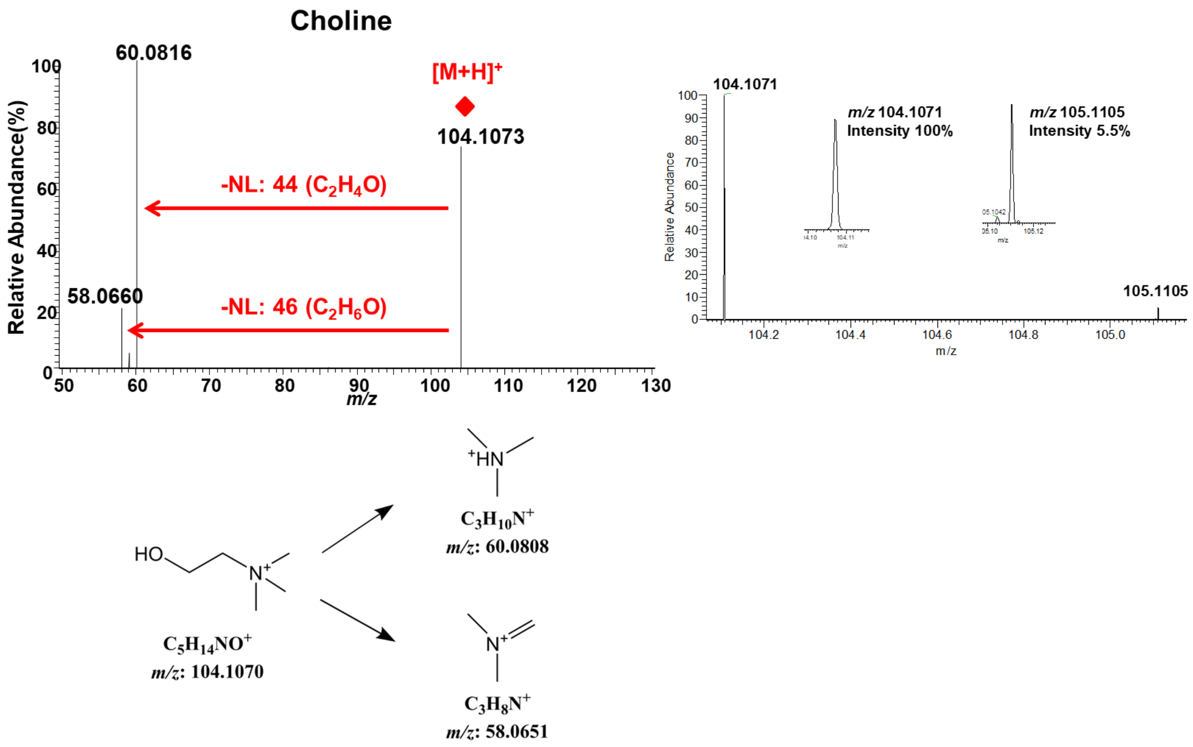

Figure S26. The isotope abundance, MS/MS spectrum and the fragmentation pathway of choline.

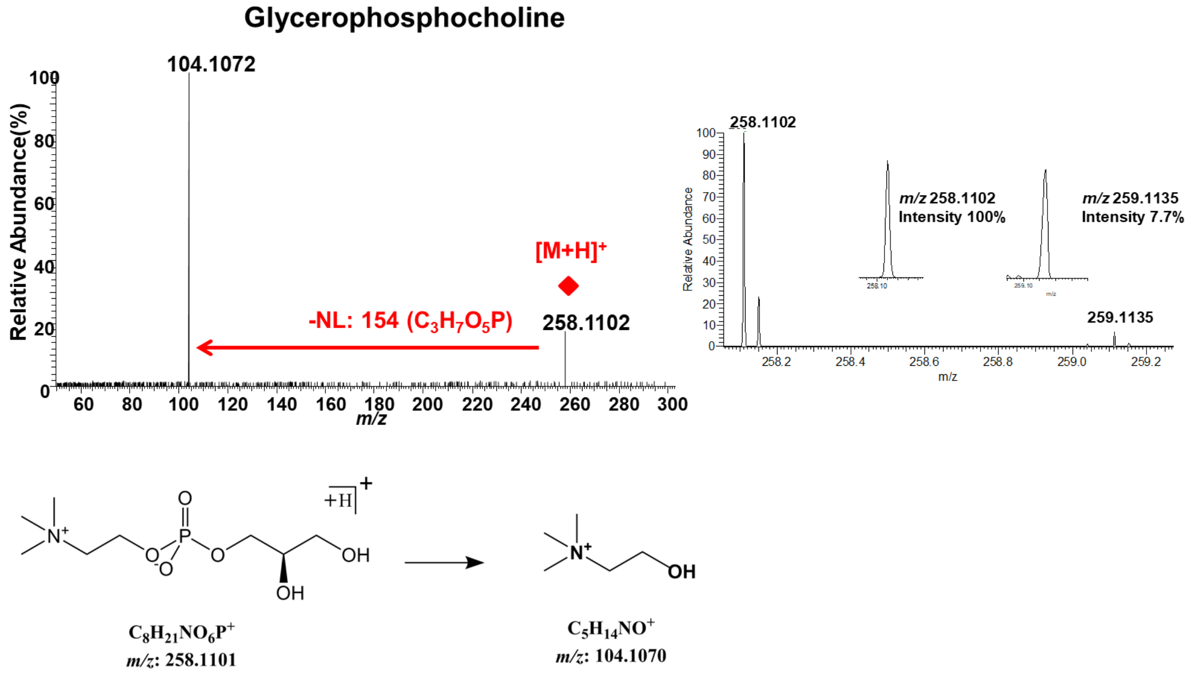

Figure S27. The isotope abundance, MS/MS spectrum and the fragmentation pathway of glycerphosphocholine.

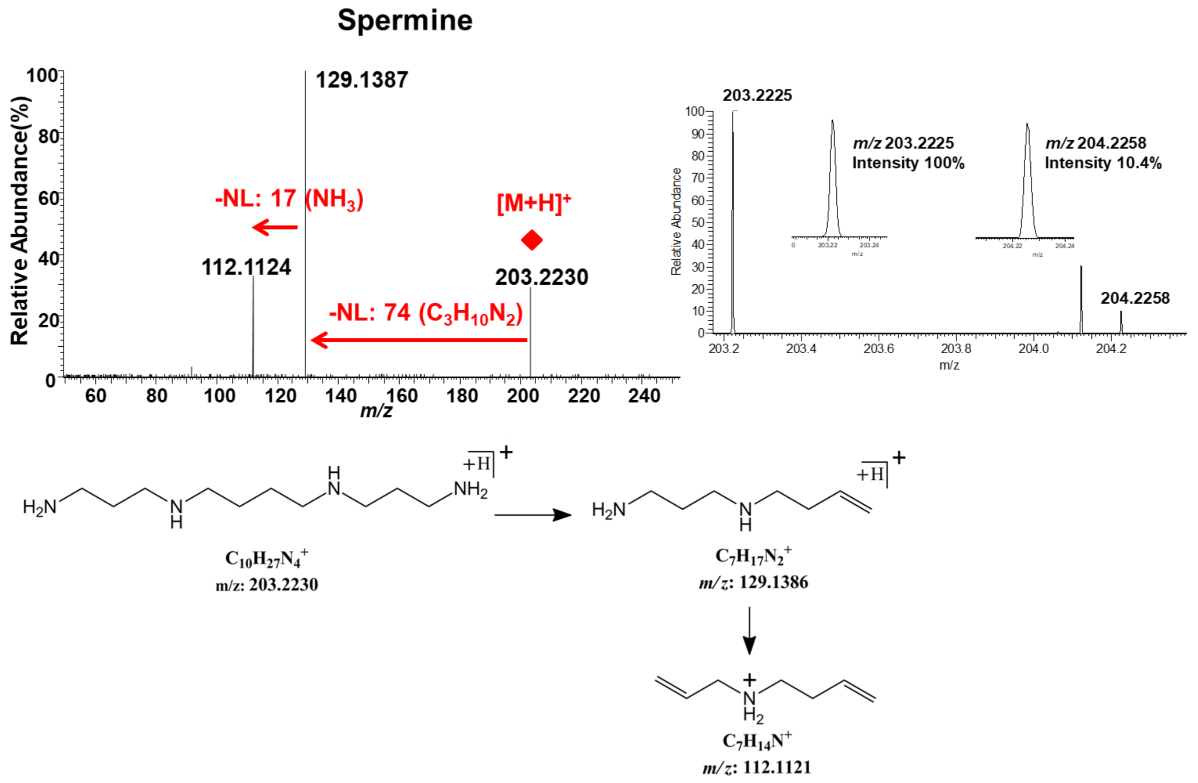

Figure S28. The isotope abundance, MS/MS spectrum and the fragmentation pathway of spermine.

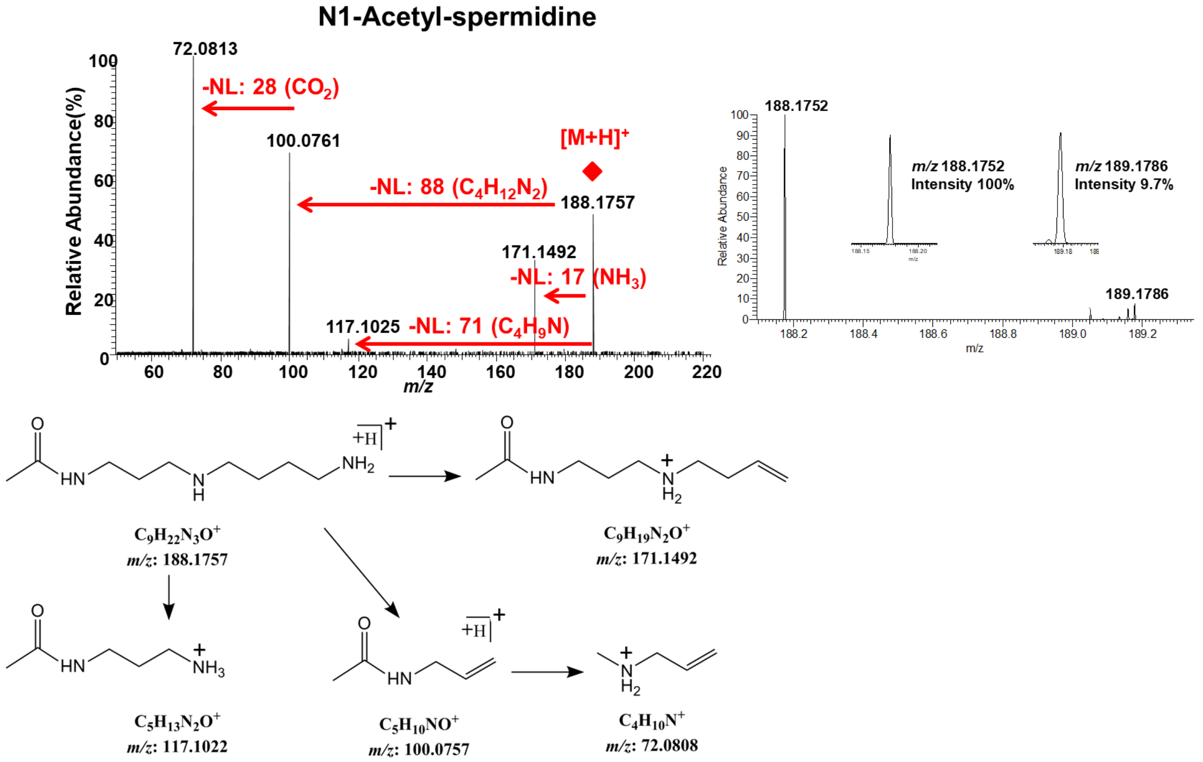

Figure S29. The isotope abundance, MS/MS spectrum and the fragmentation pathway of N1-acetyl-spermidine.

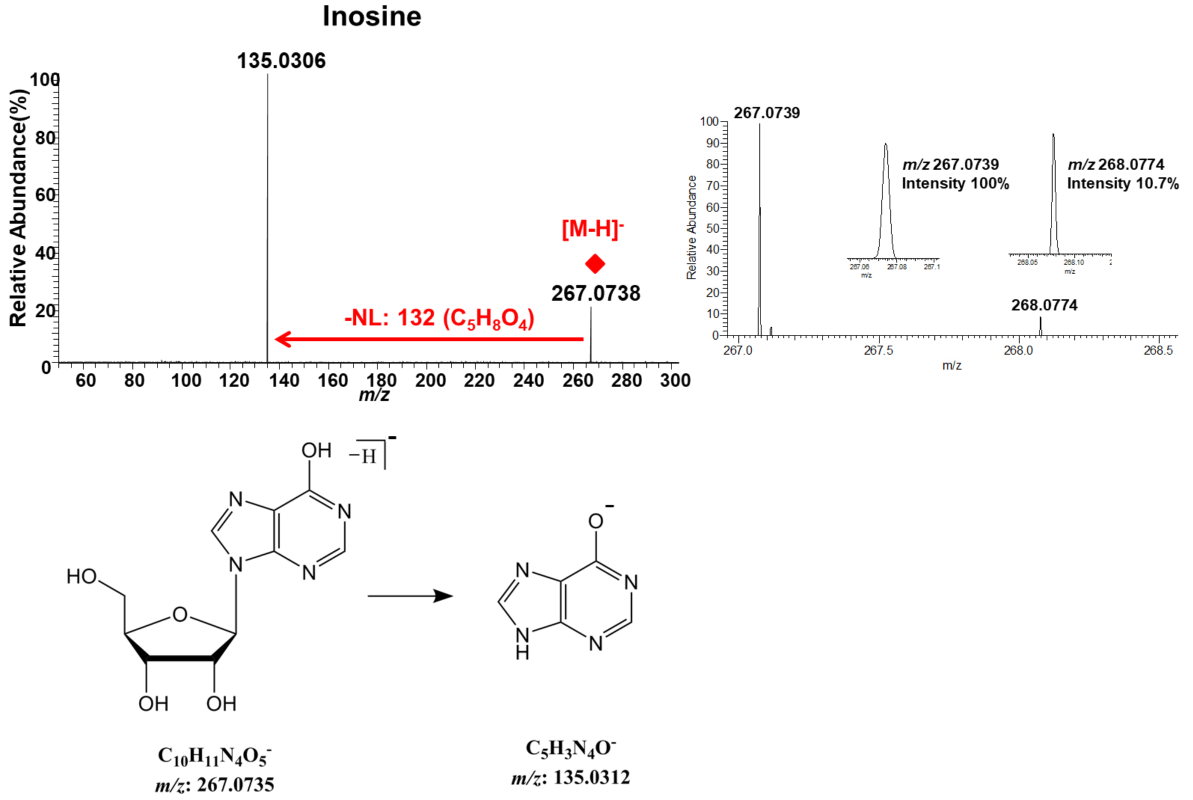

Figure S30. The isotope abundance, MS/MS spectrum and the fragmentation pathway of inosine.

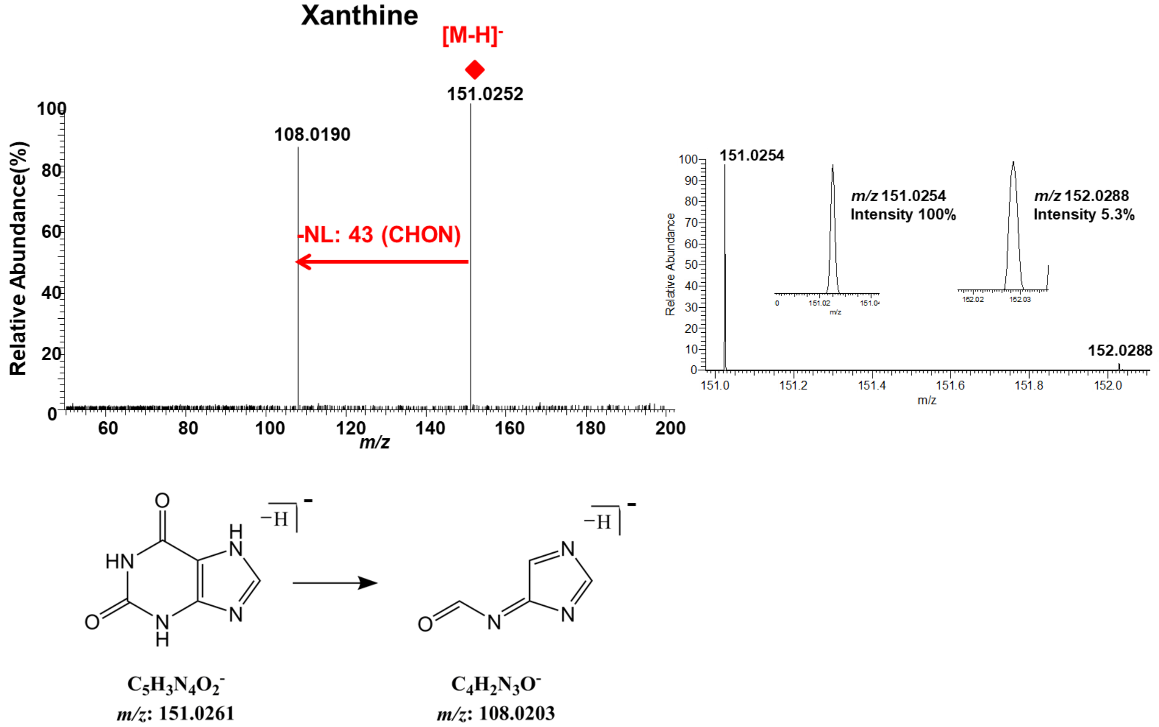

Figure S31. The isotope abundance, MS/MS spectrum and the fragmentation pathway of xanthine.

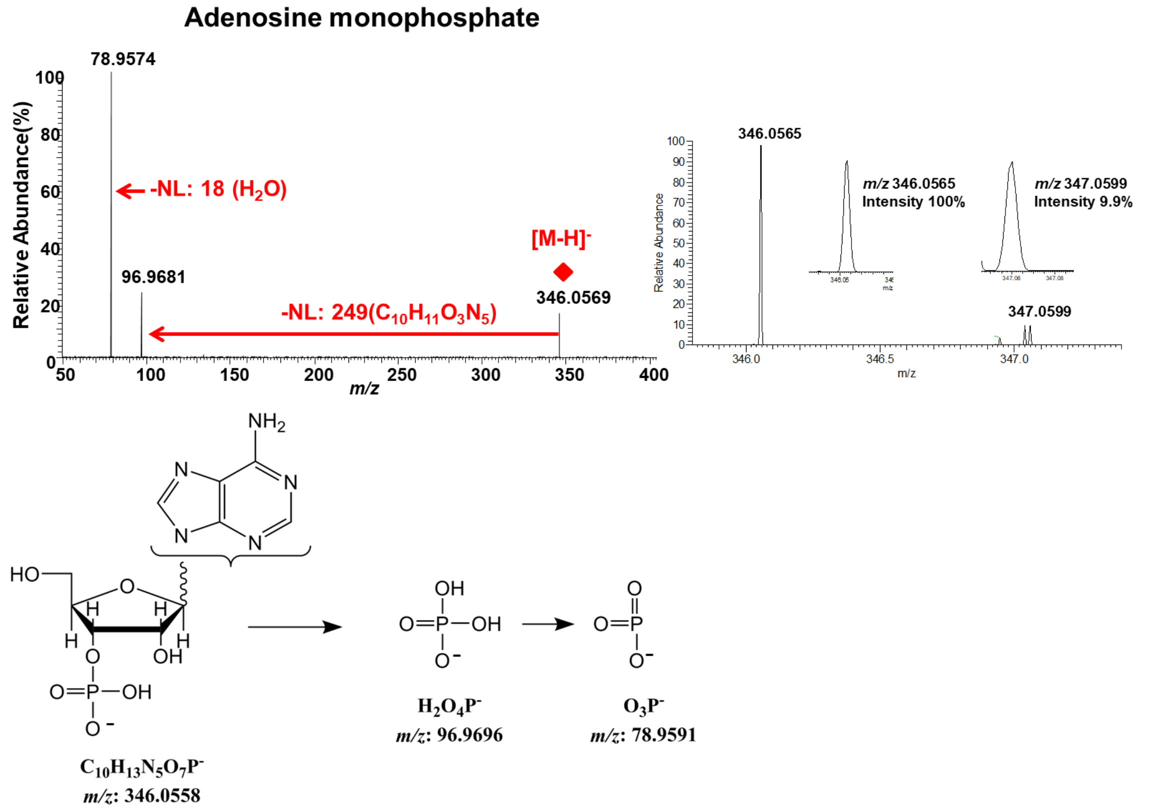

Figure S32. The isotope abundance, MS/MS spectrum and the fragmentation pathway of adenosine monophosphate.

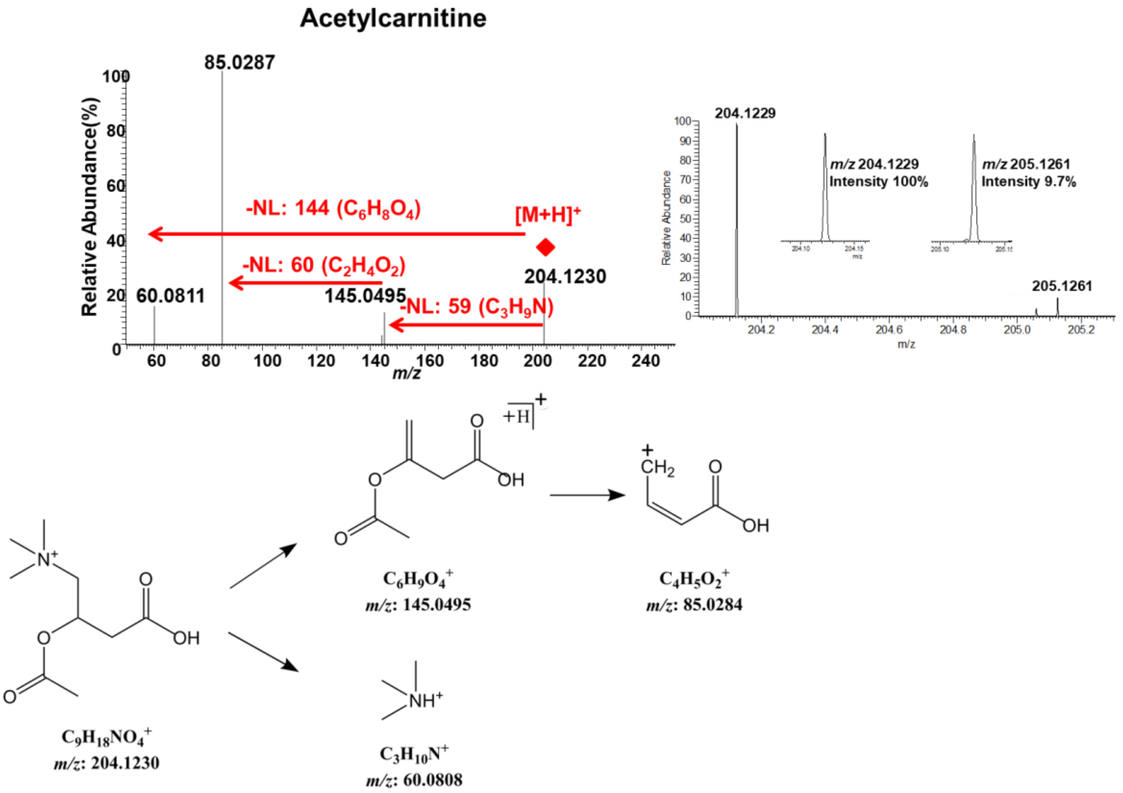

Figure S33. The isotope abundance, MS/MS spectrum and the fragmentation pathway of acetylcarnitine.

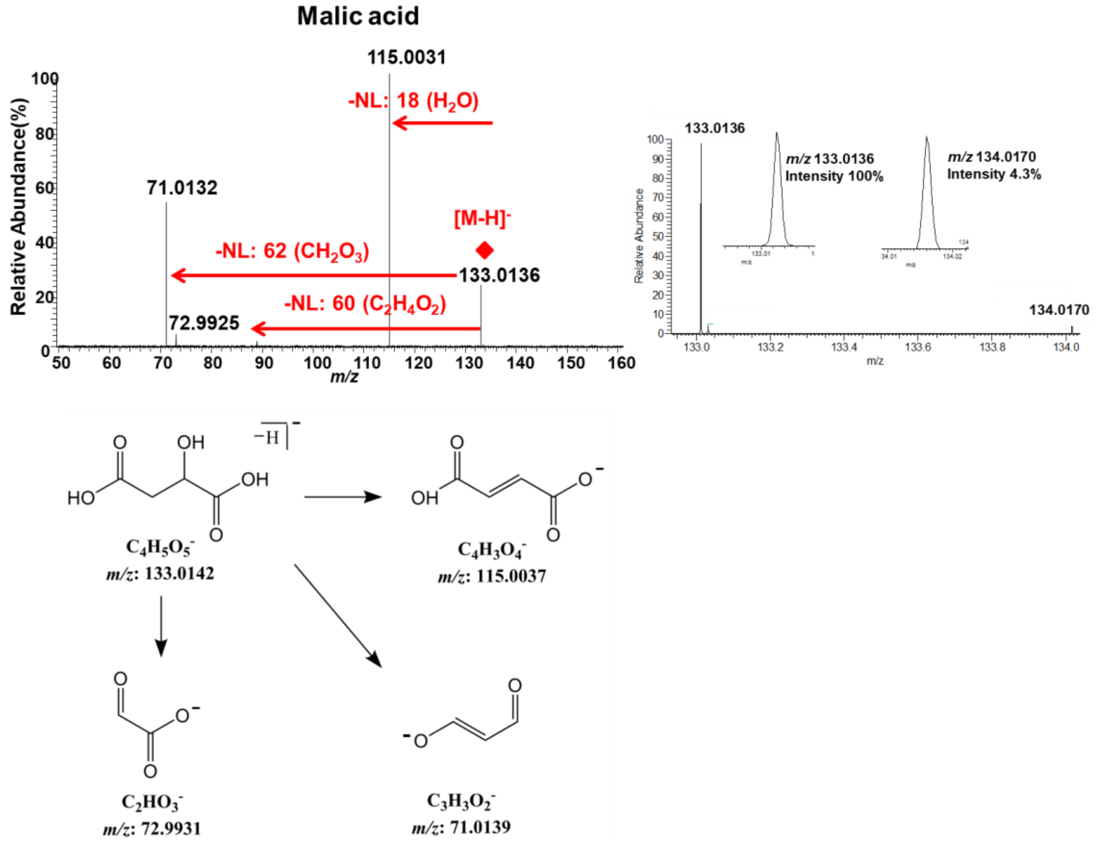

Figure S34. The isotope abundance, MS/MS spectrum and the fragmentation pathway of malic acid.

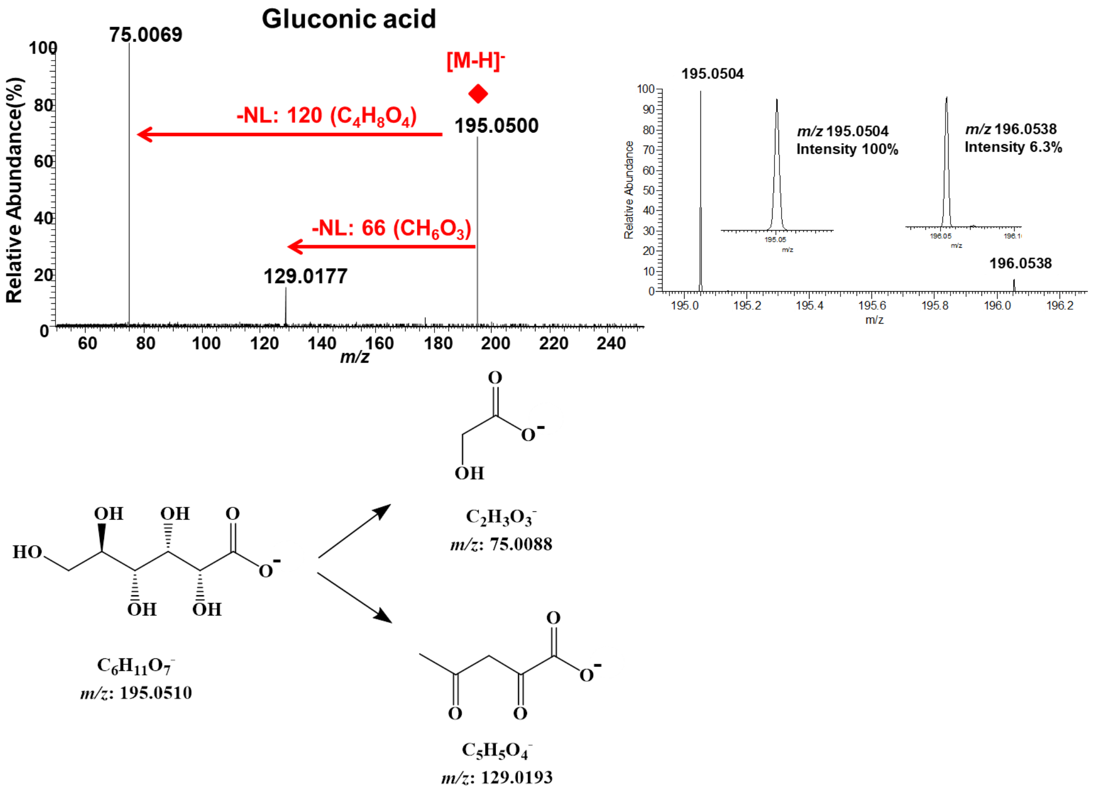

Figure S35. The isotope abundance, MS/MS spectrum and the fragmentation pathway of gluconic acid.

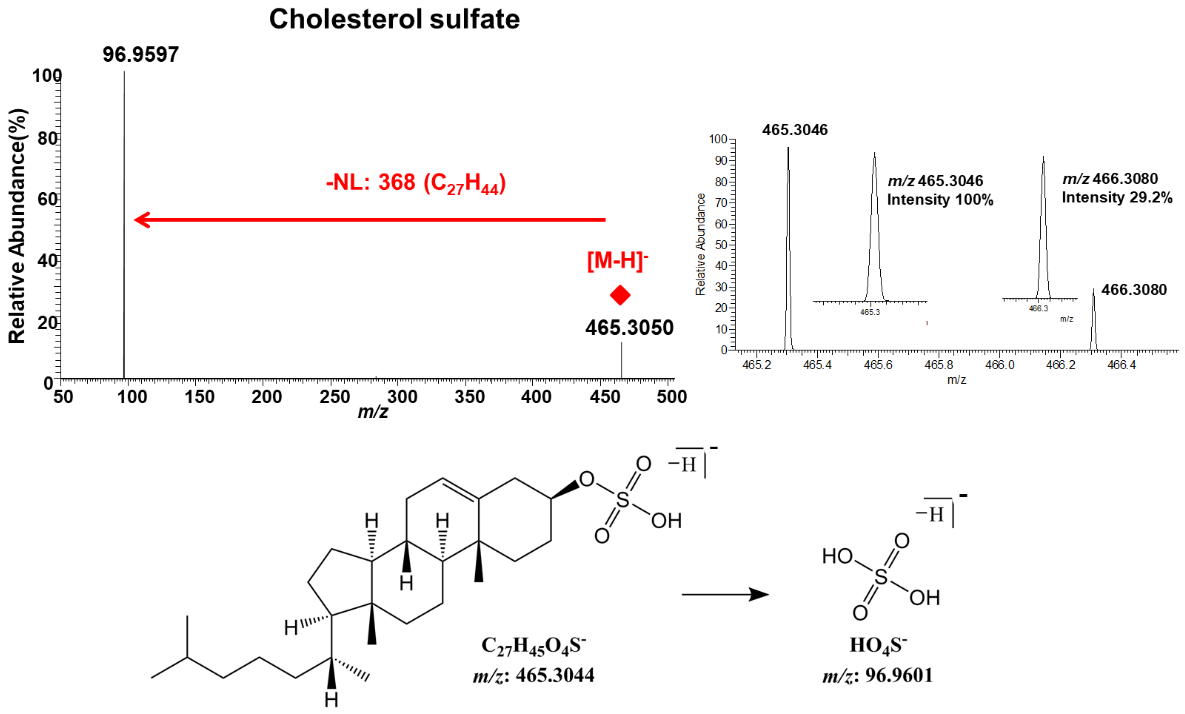

Figure S36. The isotope abundance, MS/MS spectrum and the fragmentation pathway of cholesterol sulfate.

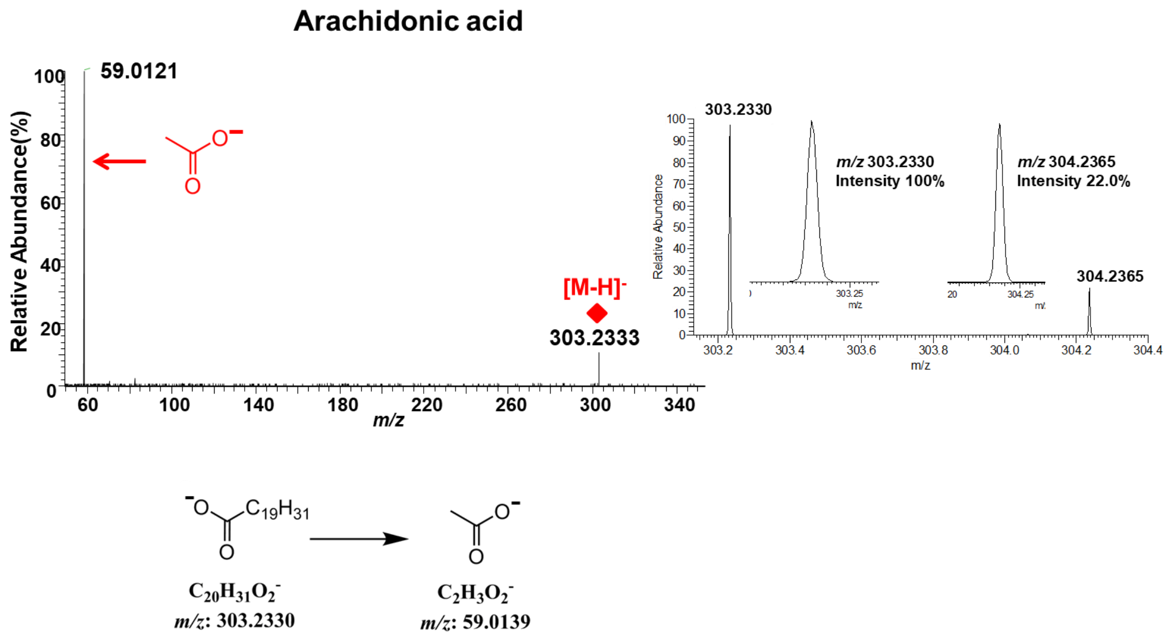

Figure S37. The isotope abundance, MS/MS spectrum and the fragmentation pathway of arachidonic acid.

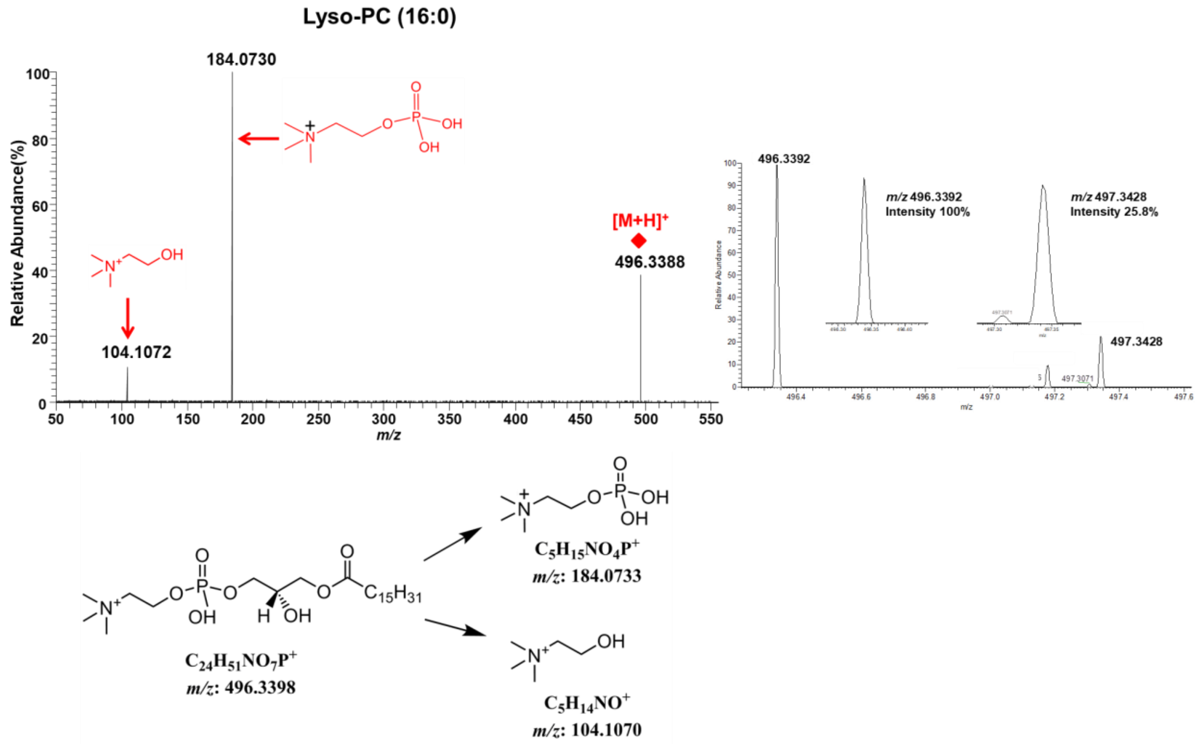

Figure S38. The isotope abundance, MS/MS spectrum and the fragmentation pathway of Lyso-PC(16:0).

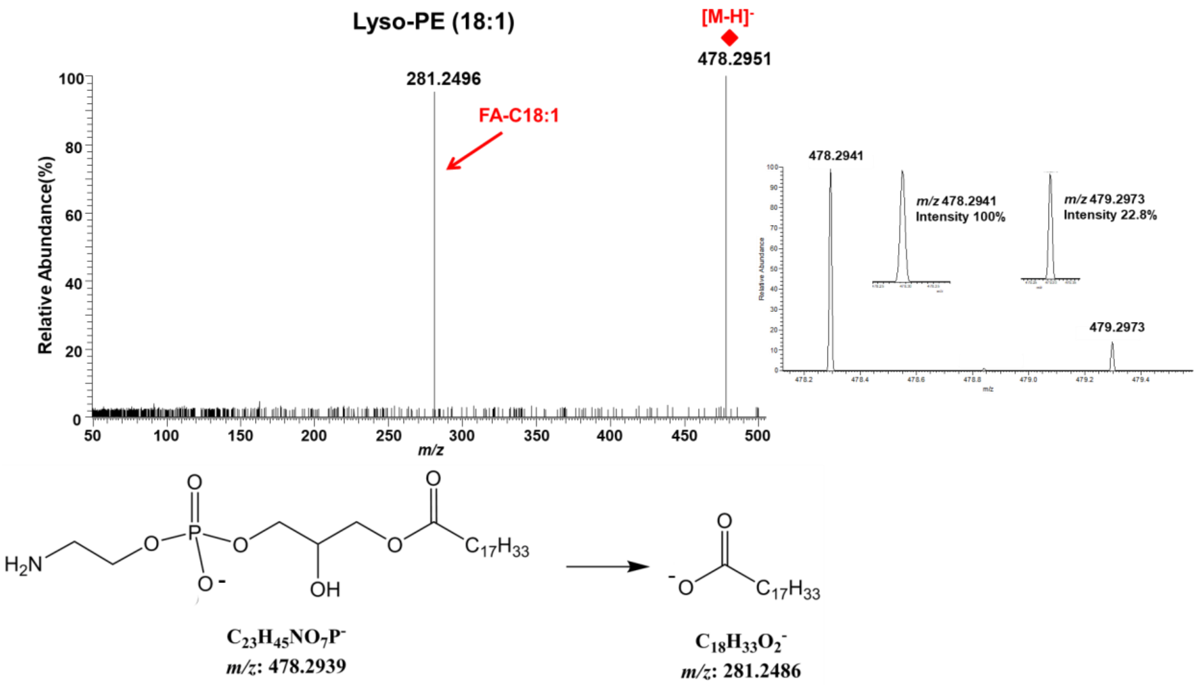

Figure S39. The isotope abundance, MS/MS spectrum and the fragmentation pathway of Lyso-PE(18:1).

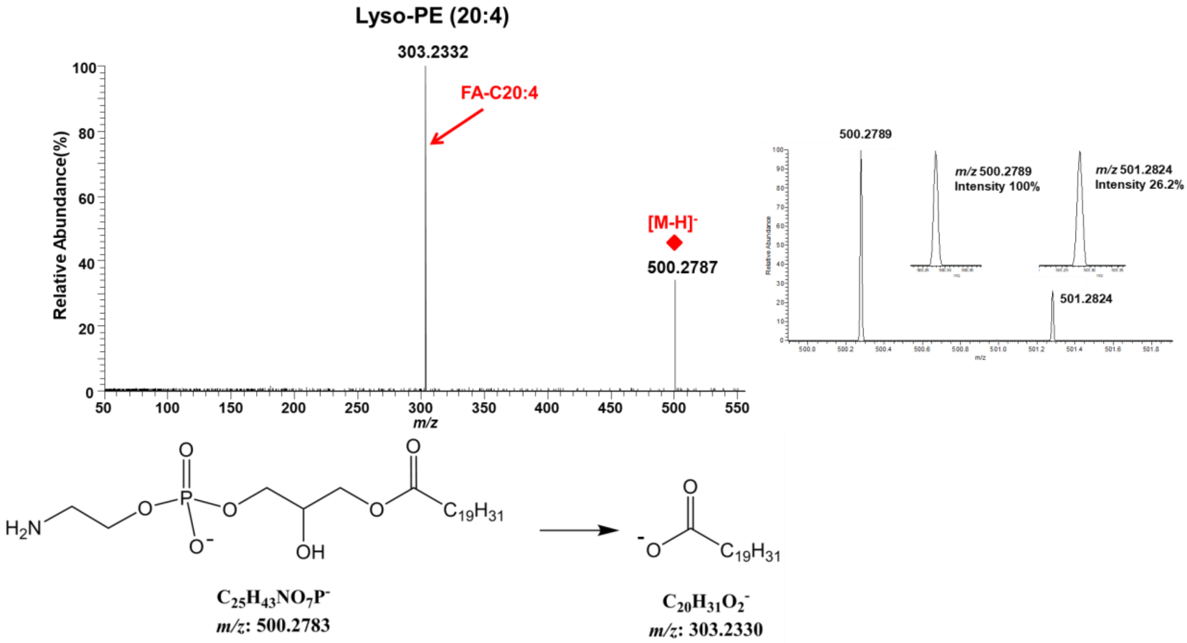

Figure S40. The isotope abundance, MS/MS spectrum and the fragmentation pathway of Lyso-PE(20:4).

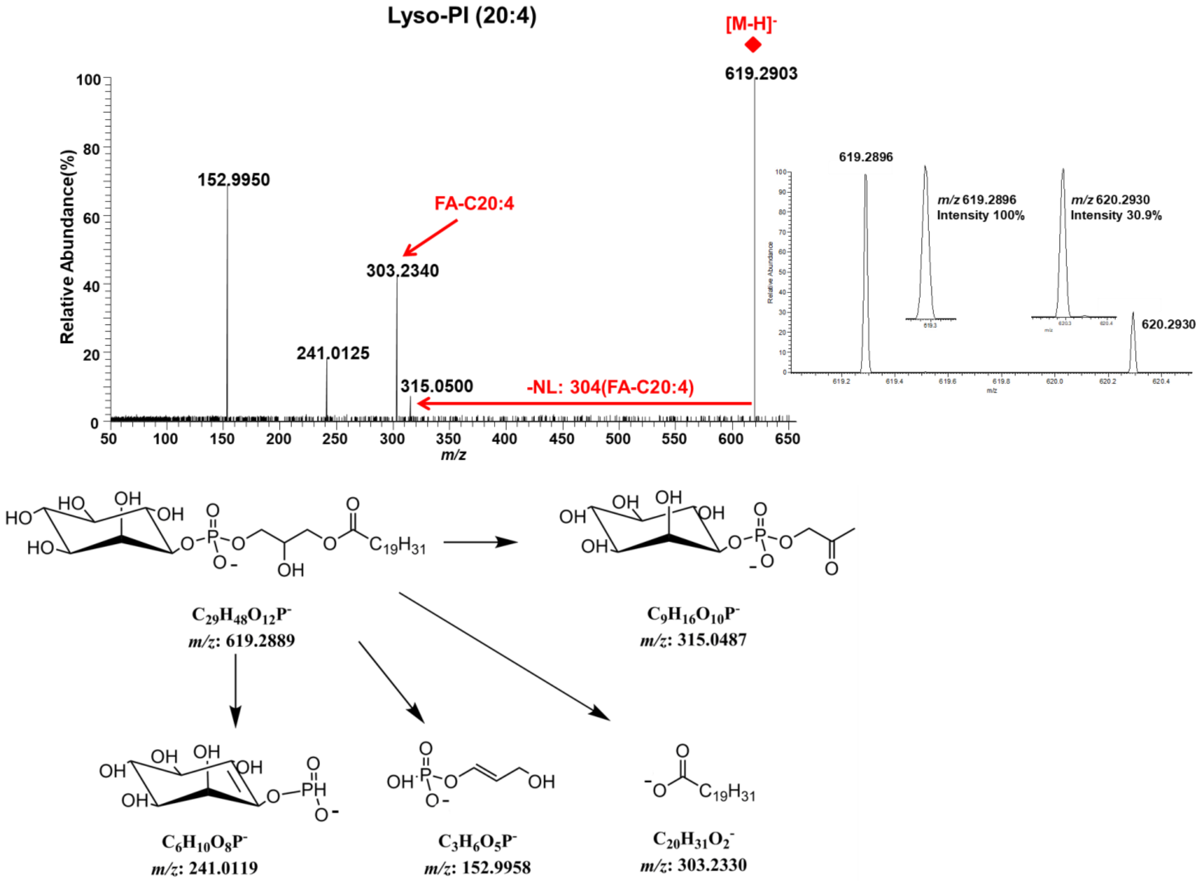

Figure S41. The isotope abundance, MS/MS spectrum and the fragmentation pathway of Lyso-PI(20:4).

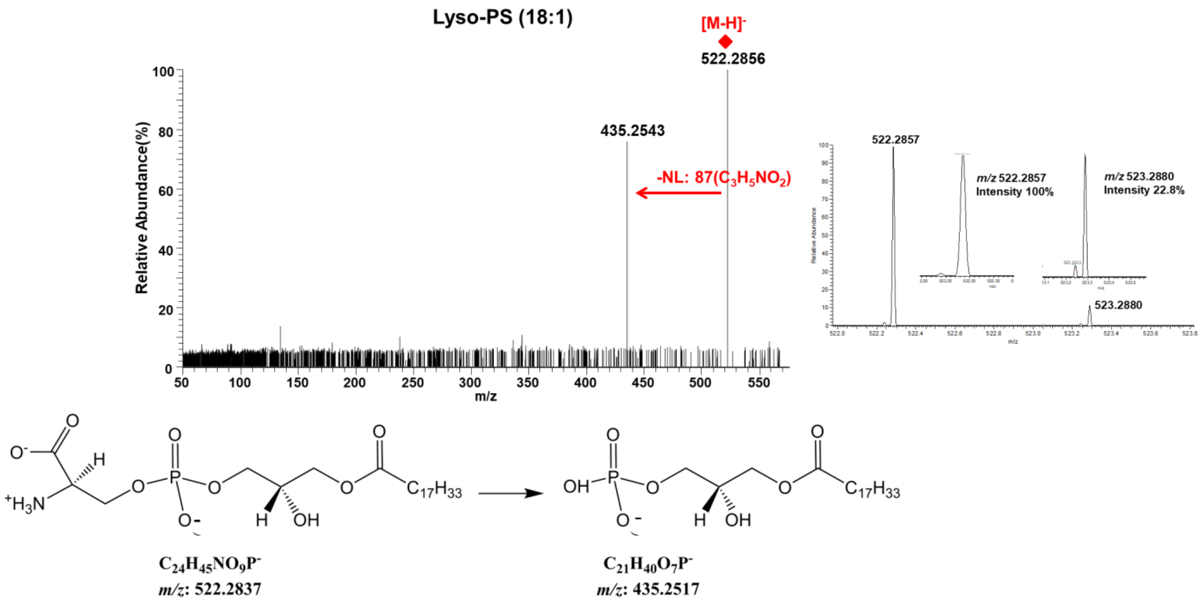

Figure S42. The isotope abundance, MS/MS spectrum and the fragmentation pathway of Lyso-PS(18:1).

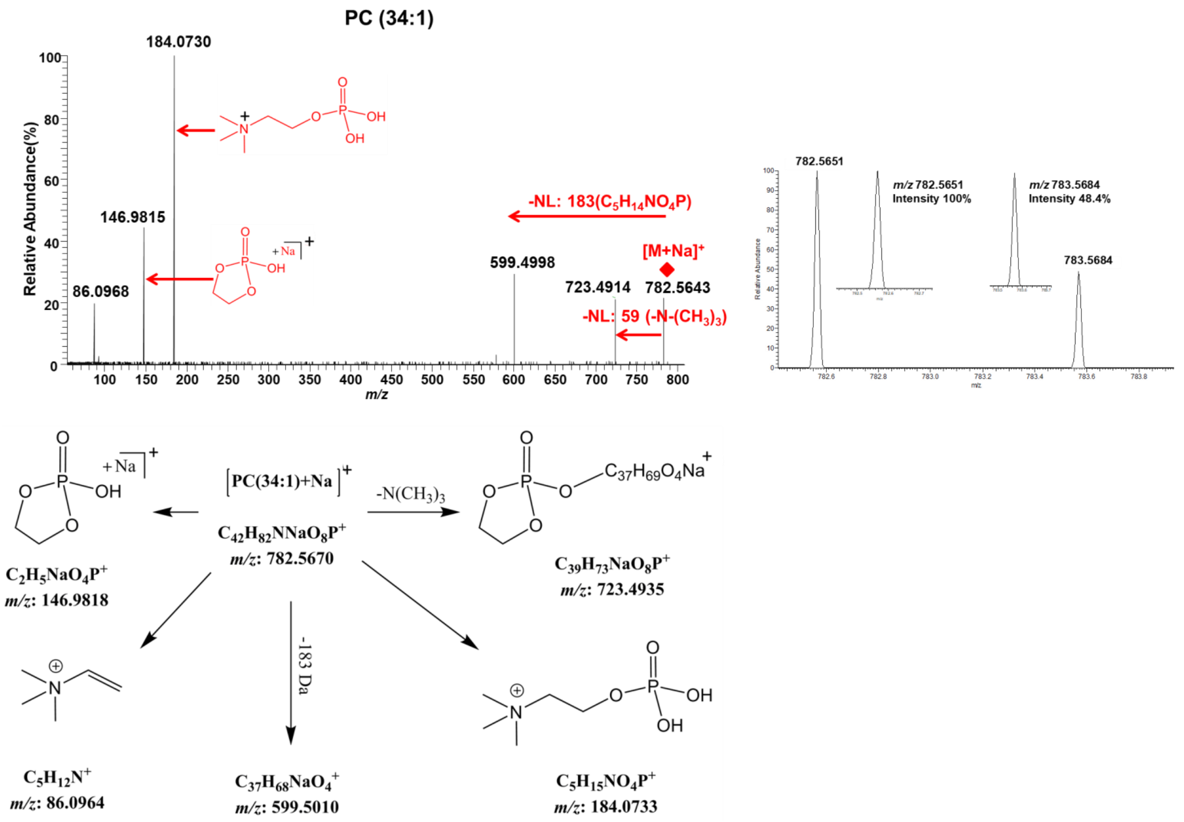

Figure S43. The isotope abundance, MS/MS spectrum and the fragmentation pathway of PC(34:1).

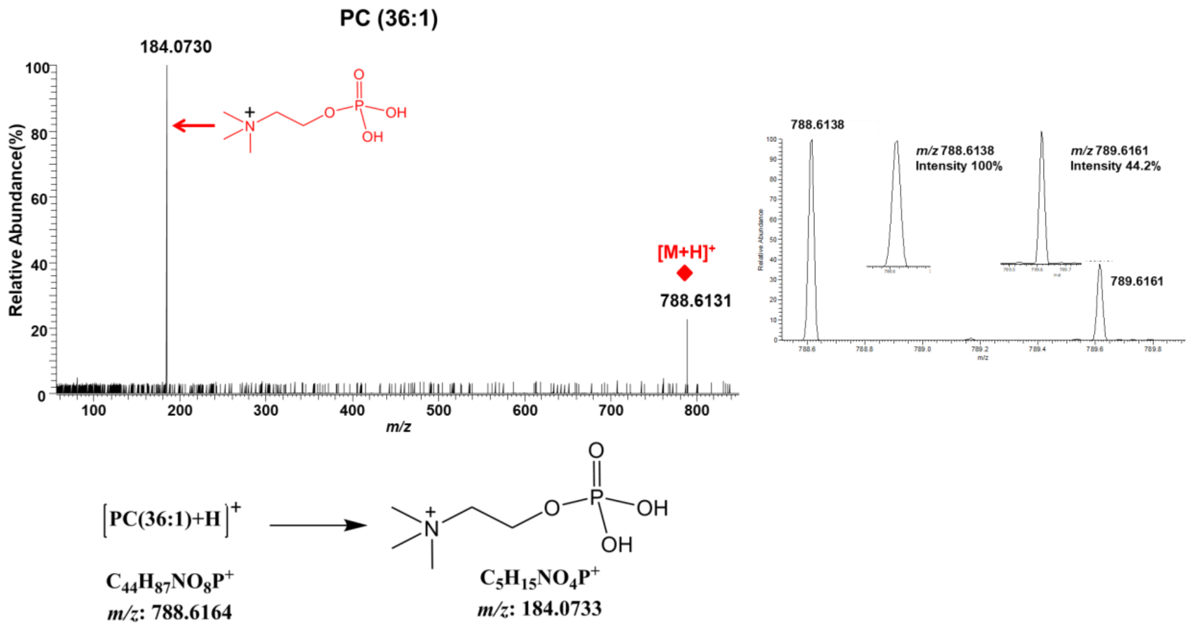

Figure S44. The isotope abundance, MS/MS spectrum and the fragmentation pathway of PC(36:1).

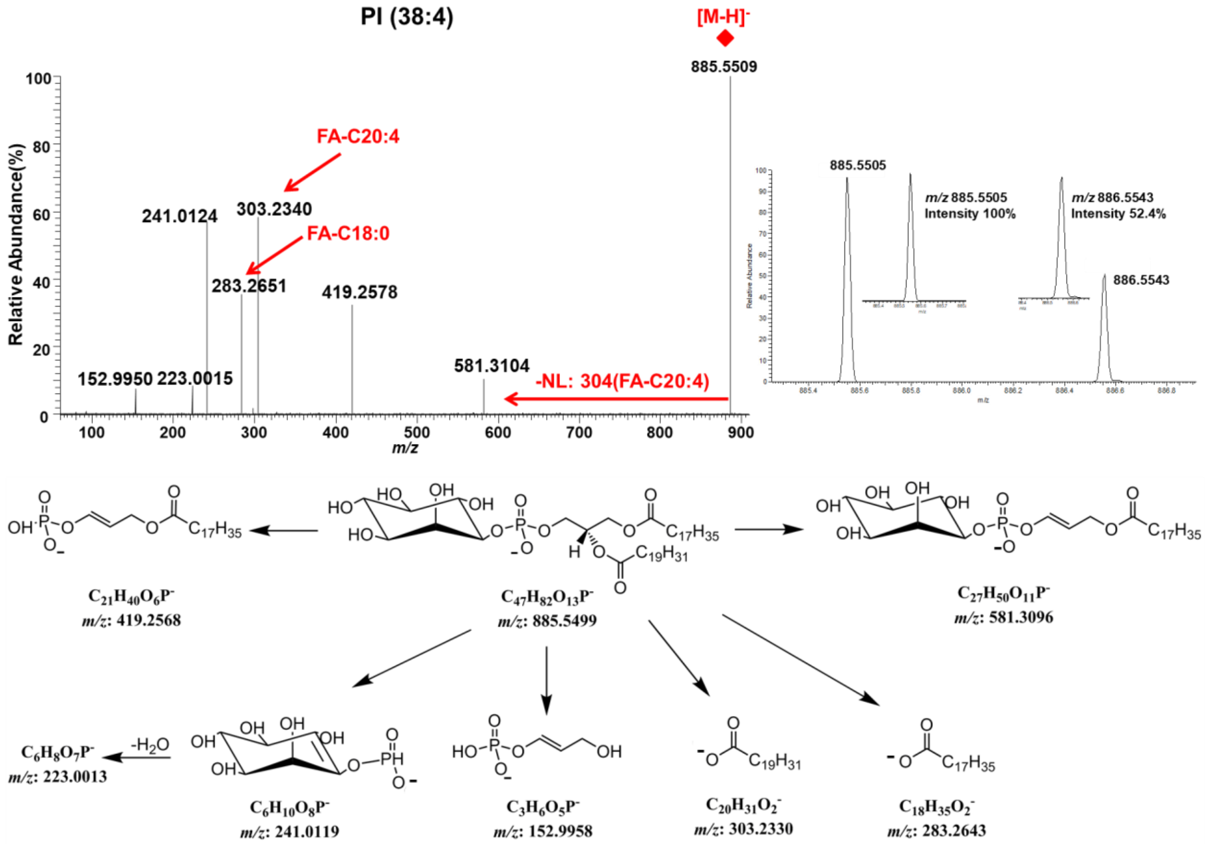

Figure S45. The isotope abundance, MS/MS spectrum and the fragmentation pathway of PI(38:4).

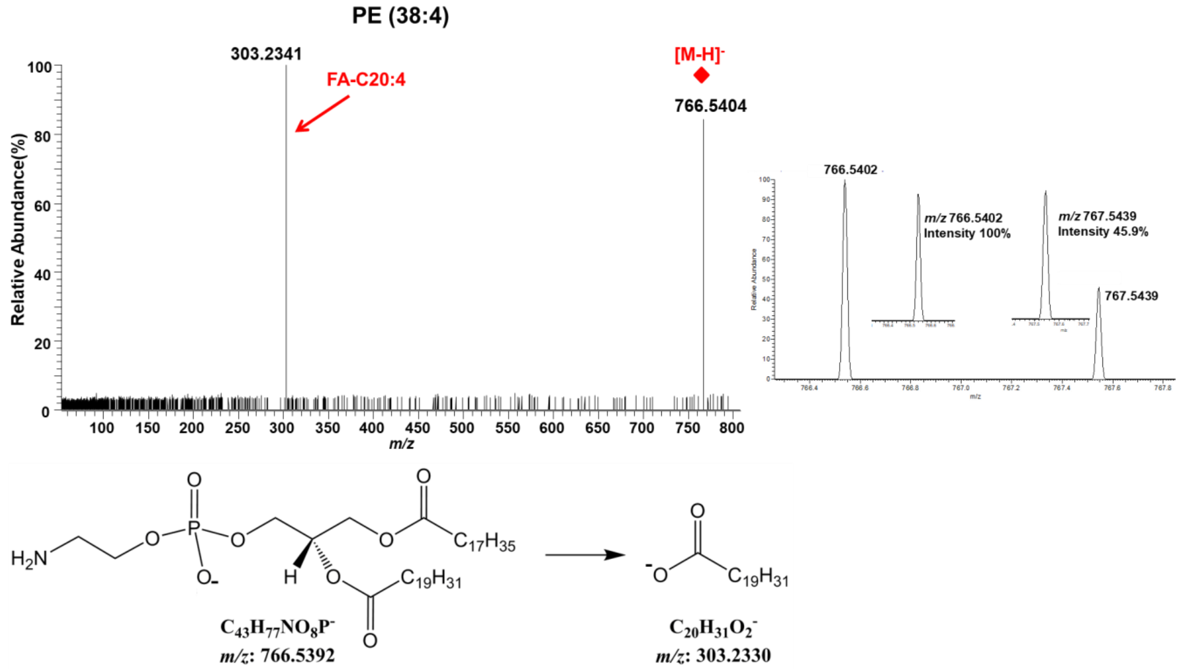

Figure S46. The isotope abundance, MS/MS spectrum and the fragmentation pathway of PE(38:4).

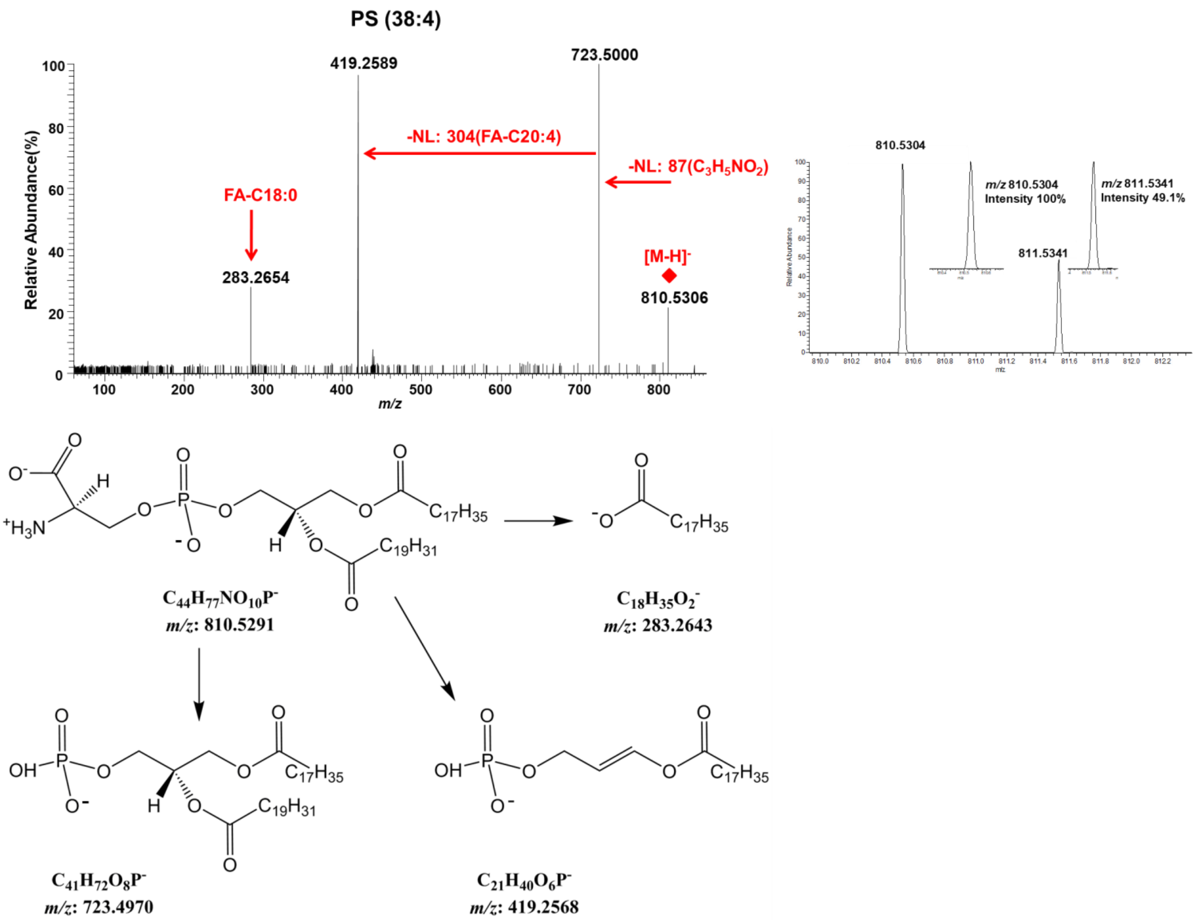

Figure S47. The isotope abundance, MS/MS spectrum and the fragmentation pathway of PS(38:4).

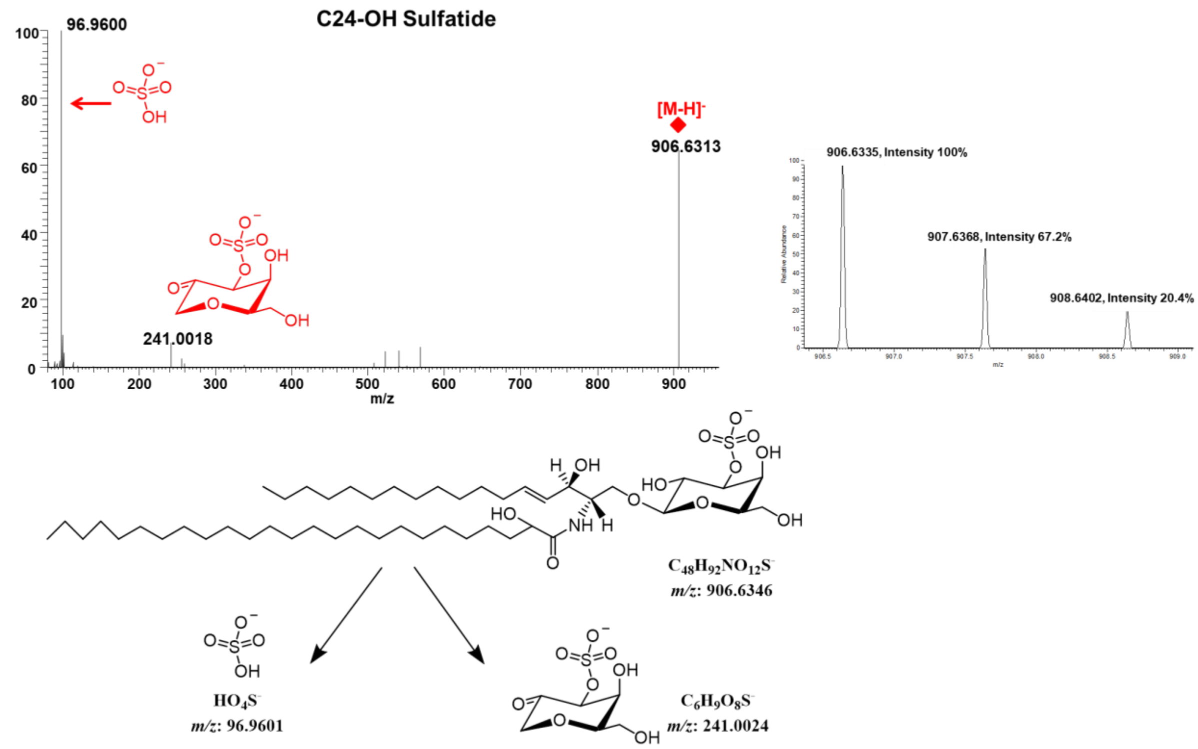

Figure S48. The isotope abundance, MS/MS spectrum and the fragmentation pathway of C24-OH sulfatide.

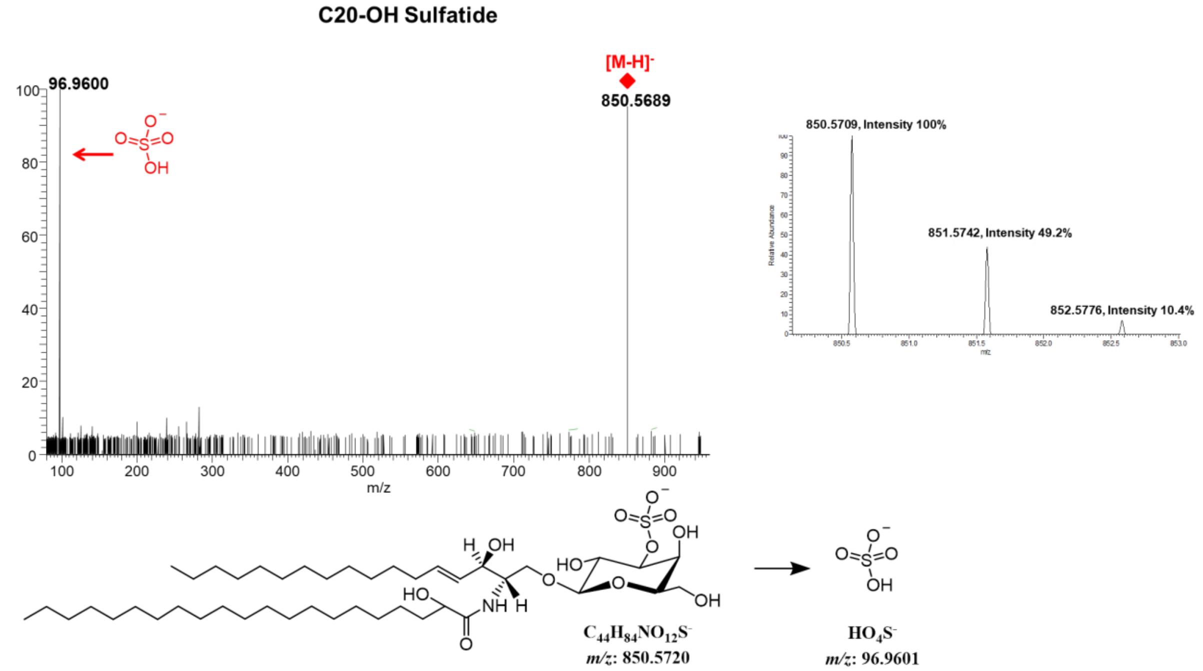

Figure S49. The isotope abundance, MS/MS spectrum and the fragmentation pathway of C20-OH sulfatide.

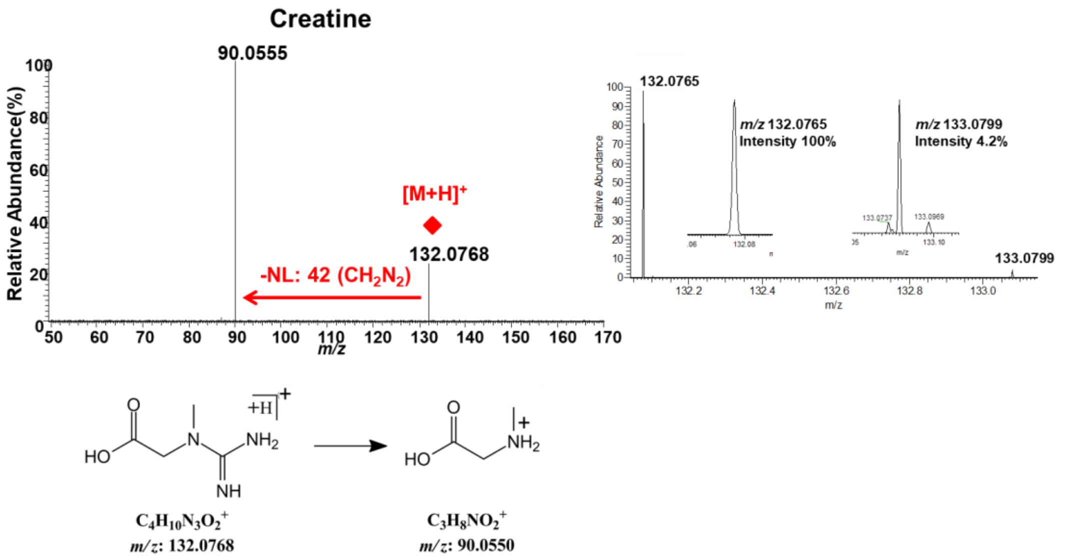

Figure S50. The isotope abundance, MS/MS spectrum and the fragmentation pathway of creatine.

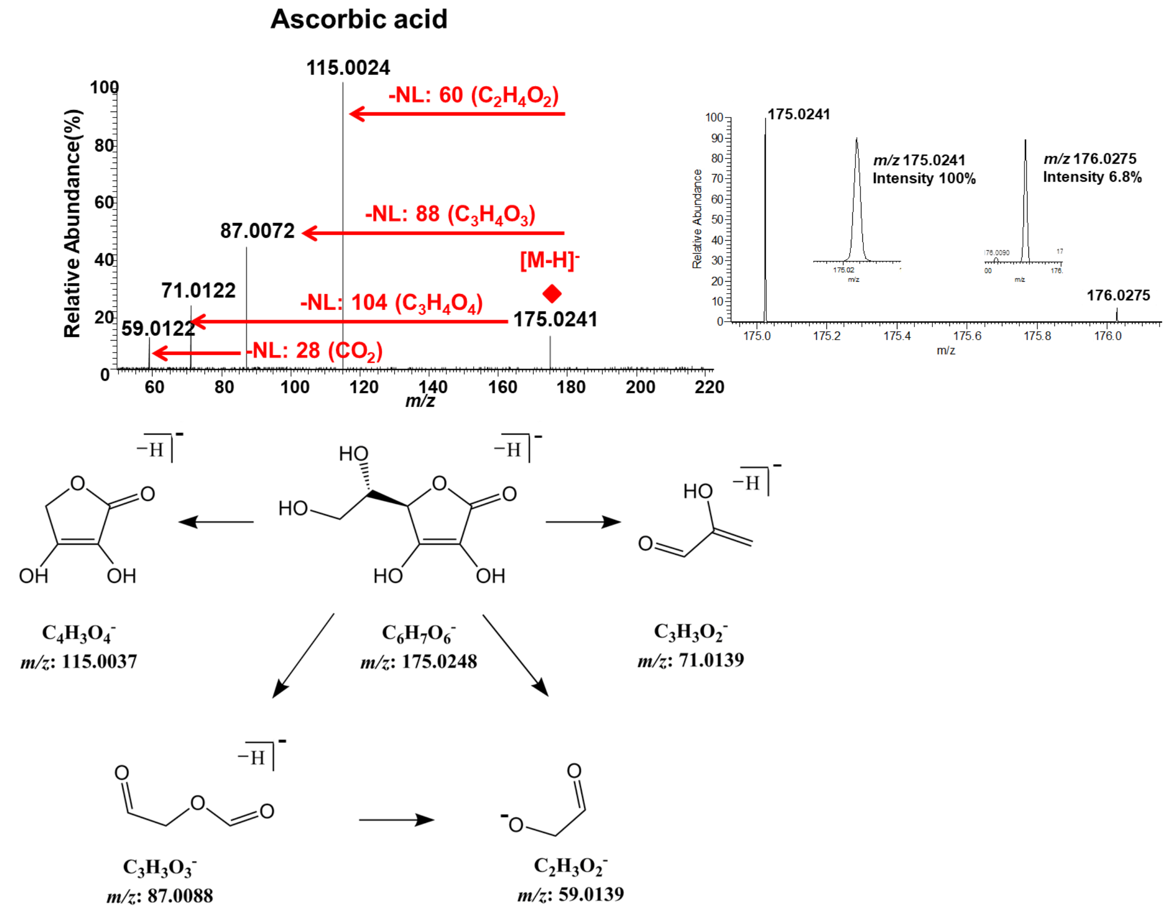

Figure S51. The isotope abundance, MS/MS spectrum and the fragmentation pathway of ascorbic acid.

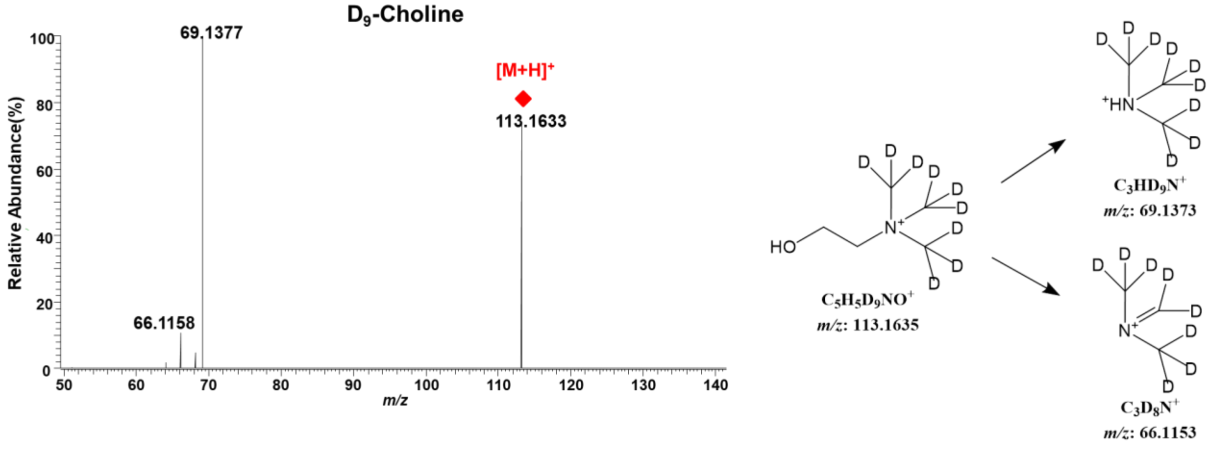

Figure S52. The isotope abundance, MS/MS spectrum and the fragmentation pathway of D_9-choline.

Table S1. Representative endogenous metabolites detected by AFADESI-MS in positive ion mode.

Species	Name	Formula	Ion type	Theoretical *m/z*	Measured *m/z*	Relative error(ppm)
Carnitine	L-Carnitine*	$C_7H_{15}NO_3$	$[M+H]^+$	162.1122	162.112	-1.23
			$[M+Na]^+$	184.0944	184.0939	-2.72
			$[M+K]^+$	200.0682	200.0678	-2.00
	L-Carnitine C2:0*	$C_9H_{17}NO_4$	$[M+H]^+$	204.123	204.1229	-0.49
			$[M+Na]^+$	226.105	226.1042	-3.54
			$[M+K]^+$	242.0789	242.078	-3.72
	L-Carnitine C3:0*	$C_{10}H_{19}NO_4$	$[M+H]^+$	218.1387	218.1384	-1.38
	L-Carnitine C4:0*	$C_{11}H_{21}NO_4$	$[M+H]^+$	232.1543	232.1541	-0.86
	L-Carnitine C5:0	$C_{12}H_{23}NO_4$	$[M+H]^+$	246.17	246.1695	-2.03
	L-Carnitine C6:0*	$C_{13}H_{25}NO_4$	$[M+H]^+$	260.1856	260.1851	-1.92
	L-Carnitine C8:0	$C_{15}H_{29}NO_4$	$[M+H]^+$	288.2169	288.217	0.35
	L-Carnitine C10:0	$C_{17}H_{33}NO_4$	$[M+H]^+$	316.2483	316.2475	-2.53
	L-Carnitine C12:0*	$C_{19}H_{37}NO_4$	$[M+H]^+$	344.2795	344.2782	-3.78
	L-Carnitine C14:0*	$C_{21}H_{41}NO_4$	$[M+H]^+$	372.3108	372.3093	-4.03
	L-Carnitine C16:1*	$C_{23}H_{43}NO_4$	$[M+H]^+$	398.3265	398.3251	-3.51
	L-Carnitine C16:0*	$C_{23}H_{45}NO_4$	$[M+H]^+$	400.3421	400.3407	-3.50
	L-Carnitine C18:1*	$C_{25}H_{47}NO_4$	$[M+H]^+$	426.3578	426.3563	-3.52
	L-Carnitine C18:2*	$C_{25}H_{35}NO_4$	$[M+H]^+$	424.3421	424.3407	-3.30
	L-Carnitine C18:0*	$C_{25}H_{39}NO_4$	$[M+H]^+$	428.3734	428.3725	-2.10
Choline	Choline*	$C_5H_{13}NO$	$[M+H]^+$	104.107	104.1071	0.96
	Acetyl choline*	$C_7H_{15}NO_2$	$[M+H]^+$	146.1175	146.1171	-2.74
	Phosphocholine*	$C_5H_{14}NO_4P$	$[M+H]^+$	184.0733	184.0728	-2.72

Species	Name	Formula	Ion type	Theoretical *m/z*	Measured *m/z*	Relative error(ppm)
Choline	Glycerophos-phocholine*	$C_8H_{20}NO_6P$	$[M+H]^+$	258.1101	258.1093	-3.10
			$[M+Na]^+$	280.092	280.0909	-3.93
			$[M+K]^+$	296.066	296.0649	-3.72
Polyamine	Spermine*	$C_{10}H_{26}N_4$	$[M+H]^+$	203.2228	203.2225	-1.48
	Spermidine*	$C_7H_{19}N_3$	$[M+H]^+$	146.1651	146.1648	-2.05
	Histamine*	$C_5H_9N_3$	$[M+H]^+$	112.0869	112.087	0.89
	Methyl-histamine*	$C_6H_{11}N_3$	$[M+H]^+$	126.1026	126.1024	-1.59
	N1-Acetyl-spermidine*	$C_9H_{21}N_3O$	$[M+H]^+$	188.1757	188.1752	-2.66
	N1,N8-Diacetyl-spermidine*	$C_{11}H_{23}N_3O_2$	$[M+H]^+$	230.1863	230.1856	-3.04
Amino-acid	Lysine*	$C_6H_{14}N_2O_2$	$[M+H]^+$	147.1128	147.1124	-2.72
	Arginine*	$C_6H_{14}N_4O_2$	$[M+H]^+$	175.119	175.1185	-2.86
	Histidine	$C_6H_9N_3O_2$	$[M+H]^+$	156.0768	156.0763	-3.20
	Glutamine*	$C_5H_{10}N_2O_3$	$[M+H]^+$	147.0764	147.076	-2.72
			$[M+Na]^+$	169.0584	169.0579	-2.96
	Proline*	$C_5H_9NO_2$	$[M+H]^+$	116.0706	116.0706	0.00
	Phenylalanine	$C_9H_{11}NO_2$	$[M+H]^+$	166.0863	166.0859	-2.14
	Alanine	$C_3H_7NO_2$	$[M+H]^+$	90.055	90.0552	2.22
	Tyrosine	$C_6H_{11}NO_3$	$[M+H]^+$	182.0812	182.0807	-2.75
	Iso/Leucine	$C_6H_{13}NO_2$	$[M+H]^+$	132.1019	132.1017	-1.51

(continued)

Species	Name	Formula	Ion type	Theoretical *m/z*	Measured *m/z*	Relative error(ppm)
**Amino-acid**	Dimethylarginine*	$C_8H_{18}N_4O_2$	$[M+H]^+$	203.1503	203.1497	-2.95
**Lyso PC**	Lyso PC(16:0)*	$C_{24}H_{50}NO_7P$	$[M+H]^+$	496.3398	496.338	-3.63
			$[M+Na]^+$	518.3217	518.3201	-3.09
			$[M+K]^+$	534.2956	534.294	-2.99
	Lyso PC(18:0)	$C_{26}H_{54}NO_7P$	$[M+H]^+$	524.3711	524.3695	-3.05
			$[M+Na]^+$	546.353	546.3516	-2.56
			$[M+K]^+$	562.3269	562.325	-3.38
	Lyso PC(18:1)*	$C_{26}H_{52}NO_7P$	$[M+H]^+$	522.3554	522.3538	-3.06
			$[M+Na]^+$	544.3374	544.3357	-3.12
			$[M+K]^+$	560.3113	560.309	-4.10
	Lyso PC(20:4)*	$C_{28}H_{50}NO_7P$	$[M+Na]^+$	566.3217	566.3196	-3.71
			$[M+K]^+$	582.2956	582.2938	-3.09
**PC**	PC(32:0)	$C_{40}H_{80}NO_8P$	$[M+H]^+$	734.5694	734.5668	-3.54
			$[M+Na]^+$	756.5514	756.5499	-1.98
			$[M+K]^+$	772.5253	772.5224	-3.75
	PC(34:1)*	$C_{42}H_{82}NO_8P$	$[M+H]^+$	760.5851	760.5823	-3.68
			$[M+Na]^+$	782.567	782.5649	-2.68
			$[M+K]^+$	798.541	798.5382	-3.51
	PC(34:2)	$C_{42}H_{80}NO_8P$	$[M+H]^+$	758.5694	758.567	-3.16
			$[M+Na]^+$	780.5514	780.5489	-3.20
			$[M+K]^+$	796.5253	796.5222	-3.89
	PC(36:2)	$C_{44}H_{84}NO_8P$	$[M+H]^+$	786.6007	786.598	-3.43

Species	Name	Formula	Ion type	Theoretical *m/z*	Measured *m/z*	Relative error(ppm)
**PC**	PC(32:1)	$C_{40}H_{78}NO_8P$	$[M+Na]^+$	754.5357	754.5338	-2.52
			$[M+K]^+$	770.5097	770.5067	-3.89
	PC(36:1)*	$C_{44}H_{86}NO_8P$	$[M+Na]^+$	810.5983	810.5964	-2.34
	PC(36:2)	$C_{44}H_{84}NO_8P$	$[M+Na]^+$	808.5827	808.58	-3.34
			$[M+K]^+$	824.5566	824.5537	-3.52
	PC(38:4)	$C_{46}H_{84}NO_8P$	$[M+Na]^+$	832.5827	832.5808	-2.28
			$[M+K]^+$	848.5566	848.5534	-3.77
	PC(36:4)	$C_{44}H_{80}NO_8P$	$[M+K]^+$	820.5253	820.5222	-3.78
	PC(38:5)	$C_{46}H_{82}NO_8P$	$[M+Na]^+$	846.541	846.5375	-4.13
	PC(38:6)	$C_{46}H_{80}NO_8P$	$[M+K]^+$	844.5253	844.5217	-4.26
	PC(40:6)	$C_{48}H_{84}NO_8P$	$[M+Na]^+$	856.5827	856.5794	-3.85
			$[M+K]^+$	872.5566	872.5528	-4.36
	PC(40:4)	$C_{48}H_{88}NO_8P$	$[M+K]^+$	876.5879	876.5839	-4.56
**SM**	SM (d18:1/16:0)	$C_{39}H_{79}N_2O_6P$	$[M+K]^+$	741.5307	741.5279	-3.78
			$[M+Na]^+$	725.5568	725.5544	-3.31
**PE**	PE(36:4)	$C_{41}H_{74}NO_7P$	$[M+Na]^+$	746.5095	746.5071	-3.21
**DG**	DG(36:2)	$C_{39}H_{72}O_5$	$[M+Na]^+$	643.5272	643.525	-3.42
			$[M+K]^+$	659.5011	659.4997	-2.12
	DG(36:3)	$C_{39}H_{70}O_5$	$[M+Na]^+$	641.5115	641.5085	-4.68
			$[M+K]^+$	657.4855	657.4837	-2.74
	DG(36:4)	$C_{39}H_{68}O_5$	$[M+Na]^+$	639.4959	639.4936	-3.60
			$[M+K]^+$	655.4698	655.4675	-3.51

(continued)

Species	Name	Formula	Ion type	Theoretical *m/z*	Measured *m/z*	Relative error(ppm)	Species	Name	Formula	Ion type	Theoretical *m/z*	Measured *m/z*	Relative error(ppm)
**Others**	Betaine*	$C_5H_{11}NO_2$	$[M+H]^+$	118.0863	118.0863	0.00	**Others**	Creatinine*	$C_4H_7N_3O$	$[M+H]^+$	114.0662	114.0662	0.00
	Creatine*	$C_4H_9N_3O_2$	$[M+H]^+$	132.0768	132.0765	-2.27				$[M+Na]^+$	136.0481	136.0478	-2.21
			$[M+Na]^+$	154.0587	154.0582	-3.25				$[M+K]^+$	152.0221	152.0216	-3.29
			$[M+K]^+$	170.0326	170.0321	-2.94		Hypoxanthin*	$C_5H_4N_4O$	$[M+H]^+$	137.0458	137.0454	-2.92
	Taurine*	$C_2H_7NO_3S$	$[M+H]^+$	126.0219	126.0218	-0.79		Inosine	$C_{10}H_{12}N_4O_5$	$[M+Na]^+$	291.07	291.069	-3.44
			$[M+Na]^+$	148.0039	148.0034	-3.38		Phospho-serine*	$C_3H_8NO_6P$	$[M+Na]^+$	207.9981	207.9976	-2.40
			$[M+K]^+$	163.9778	163.9775	-1.83							

The metabolites identified by HRMS/MS were marked with *.

Table S2. Representative endogenous metabolites detected by AFADESI-MS in negative ion mode.

**Species**	**Name**	**Formula**	**Ion type**	**Theoretical *m/z***	**Measured *m/z***	**Relative error(ppm)**	**Species**	**Name**	**Formula**	**Ion type**	**Theoretical *m/z***	**Measured *m/z***	**Relative error(ppm)**
**Fatty-acid**	FA-C12:1	$C_{12}H_{22}O_2$	$[M-H]^-$	197.1547	197.1541	-3.0	**Nucleoside & Nucleotide & Nitrogen-base**	Guanosine	$C_{10}H_{13}N_5O_5$	$[M-H]^-$	282.0844	282.0849	1.77
	FA-C14:1	$C_{14}H_{26}O_2$	$[M-H]^-$	225.1860	225.1857	-1.3		Inosine *	$C_{10}H_{12}N_4O_5$	$[M-H]^-$	267.0735	267.0739	1.50
	FA-C16:1	$C_{16}H_{30}O_2$	$[M-H]^-$	253.2173	253.2175	0.8		Uridine*	$C_9H_{12}N_2O_6$	$[M-H]^-$	243.0623	243.0623	0.00
	FA-C18:1	$C_{18}H_{34}O_2$	$[M-H]^-$	281.2486	281.2490	1.4		Adenosine monophosphate*	$C_{10}H_{14}N_5O_7P$	$[M-H]^-$	346.0558	346.0565	2.02
	FA-C20:1	$C_{20}H_{38}O_2$	$[M-H]^-$	309.2799	309.2804	1.6		Inosine monophosphate	$C_{10}H_{13}N_4O_8P$	$[M-H]^-$	347.0398	347.0400	0.58
	FA-C22:1	$C_{22}H_{42}O_2$	$[M-H]^-$	337.3112	337.3119	2.1		Guanosine monophosphate*	$C_{10}H_{14}N_5O_8P$	$[M-H]^-$	362.0507	362.0512	1.38
	FA-C24:1	$C_{14}H_{26}O_2$	$[M-H]^-$	365.3425	365.3430	1.4		Uridine monophosphate*	$C_9H_{13}N_2O_9P$	$[M-H]^-$	323.0286	323.0283	-0.93
	FA-C12:0	$C_{12}H_{24}O_2$	$[M-H]^-$	199.1704	199.1696	-4.02		Cytidine monophosphate	$C_9H_{14}N_3O_8P$	$[M-H]^-$	322.0446	322.0443	-0.93
	FA-C14:0	$C_{14}H_{28}O_2$	$[M-H]^-$	227.2017	227.2013	-1.76		Hypoxanthine*	$C_5H_4N_4O$	$[M-H]^-$	135.0312	135.0306	-4.44
	FA-C16:0	$C_{16}H_{32}O_2$	$[M-H]^-$	255.2330	255.2331	0.39		Xanthine*	$C_5H_4N_4O_2$	$[M-H]^-$	151.0261	151.0254	-4.63
	FA-C18:0	$C_{18}H_{36}O_2$	$[M-H]^-$	283.2643	283.2645	0.71		Guanine	$C_5H_5N_5O$	$[M-H]^-$	150.0421	150.0415	-4.00
	FA-C20:0	$C_{20}H_{40}O_2$	$[M-H]^-$	311.2956	311.2961	1.61		Adenine	$C_5H_5N_5$	$[M-H]^-$	134.0472	134.0468	-2.98
	FA-C22:0	$C_{22}H_{44}O_2$	$[M-H]^-$	339.3269	339.3271	0.59		Uric acid*	$C_5H_4N_4O_3$	$[M-H]^-$	167.0211	167.0204	-4.19
	FA-C24:0	$C_{24}H_{48}O_2$	$[M-H]^-$	367.3582	367.3587	1.36		Uracil	$C_4H_4N_2O_2$	$[M-H]^-$	111.0200	111.0195	-4.50
	FA-C18:3	$C_{18}H_{30}O_2$	$[M-H]^-$	277.2173	277.2176	1.08	**Amino-acid**	Valine	$C_3H_{11}NO_2$	$[M-H]^-$	116.0717	116.0712	-4.31
	FA-C22:5	$C_{22}H_{34}O_2$	$[M-H]^-$	329.2486	329.2492	1.82		Leucine/ isoleucine	$C_6H_{13}NO_2$	$[M-H]^-$	130.0874	130.0868	-4.61
	FA-C22:6*	$C_{22}H_{32}O_2$	$[M-H]^-$	327.2330	327.2335	1.53		Proline	$C_5H_9NO_2$	$[M-H]^-$	114.0561	114.0557	-3.51
	FA-C20:5*	$C_{20}H_{30}O_2$	$[M-H]^-$	301.2173	301.2177	1.33		Phenylalanine*	$C_9H_{11}NO_2$	$[M-H]^-$	164.0706	164.0703	-1.86
	FA-C18:2	$C_{18}H_{32}O_2$	$[M-H]^-$	279.2330	279.2333	1.07		Tryptophan	$C_{11}H_{12}N_2O_2$	$[M-H]^-$	203.0826	203.0819	-3.45
	FA-C20:4*	$C_{20}H_{32}O_2$	$[M-H]^-$	303.2330	303.2334	1.32		Serine*	$C_3H_7NO_3$	$[M-H]^-$	104.0353	104.0348	-4.81

(continued)

Species	Name	Formula	Ion type	Theoretical *m/z*	Measured *m/z*	Relative error(ppm)	Species	Name	Formula	Ion type	Theoretical *m/z*	Measured *m/z*	Relative error(ppm)
**Amino-acid**	Threonine*	$C_4H_9NO_3$	$[M-H]^-$	118.0510	118.0505	-4.24	**Lyso PE**	Lyso PE(22:6)*	$C_{27}H_{44}NO_7P$	$[M-H]^-$	524.2783	524.2785	0.38
	Asparaginate	$C_4H_8N2O_3$	$[M-H]^-$	131.0462	131.0456	-4.58		Lyso PE(22:5)	$C_{27}H_{46}NO_7P$	$[M-H]^-$	526.2939	526.2938	-0.19
	Glutamine*	$C_5H_{10}N_2O_3$	$[M-H]^-$	145.0619	145.0612	-4.83	**Lyso PG**	Lyso PG(20:4)	$C_{26}H_{45}O_9P$	$[M-H]^-$	531.2728	531.2727	-0.19
	Histidine*	$C_6H_9N_3O_2$	$[M-H]^-$	154.0622	154.0615	-4.54		Lyso PG(22:6)	$C_{28}H_{45}O_9P$	$[M-H]^-$	555.2728	555.2727	-0.18
	Aspartate*	$C_4H_7NO_4$	$[M-H]^-$	132.0302	132.0296	-4.54		Lyso PG(18:2)	$C_{24}H_{45}O_9P$	$[M-H]^-$	507.2728	507.2728	0.00
	Glutamate*	$C_5H_9NO_4$	$[M-H]^-$	146.0459	146.0452	-4.79		Lyso PG(18:1)	$C_{24}H_{47}O_9P$	$[M-H]^-$	509.2885	509.2885	0.00
	Tyrosine	$C_6H_{11}NO_3$	$[M-H]^-$	180.0666	180.0658	-4.44	**Lyso PI**	Lyso PI(16:1)	$C_{25}H_{47}O_{12}P$	$[M-H]^-$	569.2732	569.2735	0.53
	Acetyl aspartic acid*	$C_6H_9NO_5$	$[M-H]^-$	174.0408	174.0400	-4.60		Lyso PI(16:0)*	$C_{25}H_{49}O_{12}P$	$[M-H]^-$	571.2889	571.289	0.18
**Organic-acid**	Iso/Citric-acid*	$C_6H_7O_7$	$[M-H]^-$	191.0197	191.0188	-4.71		Lyso PI(18:1)	$C_{27}H_{51}O_{12}P$	$[M-H]^-$	597.3045	597.3049	0.67
	Aconitic acid	$C_6H_5O_6$	$[M-H]^-$	173.0092	173.0084	-4.62		Lyso PI(18:0)*	$C_{27}H_{53}O_{12}P$	$[M-H]^-$	599.3202	599.3208	1.00
	Succinic acid	$C_4H_5O_4$	$[M-H]^-$	117.0193	117.0188	-4.27		Lyso PI(20:4)*	$C_{29}H_{49}O_{12}P$	$[M-H]^-$	619.2889	619.2895	0.97
	Maleic acid/ Fumaric acid*	$C_4H_3O_4$	$[M-H]^-$	115.0037	115.0032	-4.35	**Lyso PS**	Lyso PS(22:6)	$C_{28}H_{44}NO_9P$	$[M-H]^-$	568.2681	568.2687	1.06
	Malic acid*	C4H_5O_5	$[M-H]^-$	133.0142	133.0136	-4.51		Lyso PS(20:4)*	$C_{26}H_{44}NO_9P$	$[M-H]^-$	544.2681	544.2681	0.00
	taurine*	$C_2H_7NO_3S$	$[M-H]^-$	124.0074	124.0068	-4.84		Lyso PS(18:1)*	$C_{24}H_{46}NO_9P$	$[M-H]^-$	522.2837	522.2838	0.19
**Lyso PE**	LysoPE(16:0)*	$C_{21}H_{44}NO_7P$	$[M-H]^-$	452.2783	452.2784	0.22	**Fatty-acid hydroxy fatty-acids**	PAHSA	$C_{34}H_{66}O_4$	$[M-H]^-$	537.4888	537.4894	1.12
	LysoPE(18:0)	$C_{23}H_{48}NO_7P$	$[M-H]^-$	480.3096	480.3096	0.00		POHSA	$C_{34}H_{64}O_4$	$[M-H]^-$	535.4732	535.4733	0.19
	LysoPE(18:1)*	$C_{23}H_{46}NO_7P$	$[M-H]^-$	478.2939	478.2942	0.63		OAHSA	$C_{36}H_{68}O_4$	$[M-H]^-$	563.5045	563.5048	0.53
	PE(P-16:0/0:0)	$C_{21}H_{44}NO_6P$	$[M-H]^-$	436.2833	436.2833	0.00	**PE**	PE-(P-36:1)	$C_{41}H_{80}NO_7P$	$[M-H]^-$	728.56	728.5611	1.51
	Lyso PE(16:1)	$C_{21}H_{42}NO_7P$	$[M-H]^-$	450.2626	450.2625	-0.22		PE(36:2)	$C_{41}H_{78}NO_8P$	$[M-H]^-$	742.5392	742.5405	1.75
	Lyso PE(18:2)	$C_{23}H_{44}NO_7P$	$[M-H]^-$	476.2783	476.278	-0.63		PE(36:1)	$C_{41}H_{80}NO_8P$	$[M-H]^-$	744.5549	744.5556	0.94
	LysoPE(20:4)*	$C_{25}H_{44}NO_7P$	$[M-H]^-$	500.2783	500.2787	0.80		PE-(P-38:6)	$C_{43}H_{74}NO_7P$	$[M-H]^-$	746.513	746.5141	1.47

(continued)

Species	Name	Formula	Ion type	Theoretical *m/z*	Measured *m/z*	Relative error(ppm)
**PE**	PE(34:1)	$C_{39}H_{76}NO_8P$	$[M-H]^-$	716.5235	716.5255	2.79
	PE(P-38:4) /PE(O-38:5)	$C_{43}H_{78}NO_7P$	$[M-H]^-$	750.5443	750.5451	1.07
	PE(P-38:2)/ PE(O-38:3)	$C_{43}H_{82}NO_7P$	$[M-H]^-$	754.5756	754.5767	1.46
	PE(38:6)*	$C_{43}H_{74}NO_8P$	$[M-H]^-$	762.5079	762.5086	0.92
	PE(38:4)*	$C_{43}H_{78}NO_8P$	$[M-H]^-$	766.5392	766.5404	1.57
	PE(P-40:6)	$C_{45}H_{78}NO_7P$	$[M-H]^-$	774.5443	774.5451	1.03
	PE(P-40:4)	$C_{45}H_{82}NO_7P$	$[M-H]^-$	778.5756	778.5763	0.90
	PE(40:4)	$C_{45}H_{82}NO_8P$	$[M-H]^-$	794.5705	794.5726	2.64
	PE(46:3)	$C_{51}H_{96}NO_8P$	$[M+Cl^-$	916.6568	916.657	0.22
**PA**	PA(34:1)	$C_{37}H_{71}O_8P$	$[M-H]^-$	678.4814	678.4823	1.33
	PA(P-34:0)/ PA(O-34:1)	$C_{37}H_{73}O_7P$	$[M-H]^-$	659.5021	659.5032	1.67
	PA(P-34:1)/ PA(O-34:2)	$C_{37}H_{71}O_7P$	$[M-H]^-$	657.4865	657.4874	1.37
	PA(P-34:2)/ PA(O-34:3)	$C_{37}H_{69}O_7P$	$[M-H]^-$	655.4708	655.4741	5.03
	PA(P-36:2)/ PA(O-36:3)	$C_{39}H_{73}O_7P$	$[M-H]^-$	683.5021	683.5026	0.73
	PA(P-36:1)/ PA(O-36:2)	$C_{39}H_{75}O_7P$	$[M-H]^-$	685.5178	685.5185	1.02

Species	Name	Formula	Ion type	Theoretical *m/z*	Measured *m/z*	Relative error(ppm)
**PA**	PA(36:1)	$C_{39}H_{75}O_8P$	$[M-H]^-$	701.5127	701.5107	-2.85
**PS**	PS(36:2)*	$C_{42}H_{78}NO_{10}P$	$[M-H]^-$	786.5291	786.5303	1.53
	PS(36:1)*	$C_{42}H_{80}NO_{10}P$	$[M-H]^-$	788.5447	788.5461	1.78
	PS(34:1)	$C_{40}H_{76}NO_{10}P$	$[M-H]^-$	760.5134	760.5149	1.97
	PS-(38:4)*	$C_{44}H_{78}NO_{10}P$	$[M-H]^-$	810.5291	810.5304	1.60
	PS-(38:2)	$C_{44}H_{82}NO_{10}P$	$[M-H]^-$	814.5604	814.5616	1.47
	PS-(38:1)	$C_{44}H_{84}NO_{10}P$	$[M-H]^-$	816.576	816.5775	1.84
	PS-(40:6)*	$C_{46}H_{78}NO_{10}P$	$[M-H]^-$	834.5291	834.5306	1.80
	PS-(40:4)	$C_{46}H_{82}NO_{10}P$	$[M-H]^-$	838.5604	838.5624	2.39
	PS-(40:1)	$C_{46}H_{88}NO_{10}P$	$[M-H]^-$	844.6073	844.6075	0.24
	PS-(40:2)	$C_{46}H_{86}NO_{10}P$	$[M-H]^-$	842.5917	842.5923	0.71
	PS-(42:2)	$C_{48}H_{90}NO_{10}P$	$[M-H]^-$	870.623	870.6242	1.38
	PS(42:1)	$C_{48}H_{92}NO_{10}P$	$[M-H]^-$	872.6386	872.6377	-1.03
	PS(44:12)	$C_{50}H_{74}NO_{10}P$	$[M-H]^-$	878.4978	878.499	1.37
	PS(44:10)	$C_{50}H_{78}NO_{10}P$	[M-H]-	882.5291	882.5278	-1.47
	PG(36:4)	$C_{42}H_{75}O_{10}P$	$[M-H]^-$	769.5025	769.5039	1.82
**PG**	PG(34:1)	$C_{40}H_{77}O_{10}P$	$[M-H]^-$	747.5182	747.5193	1.47
	PG-(38:4)	$C_{44}H_{79}O_{10}P$	$[M-H]^-$	797.5338	797.5339	0.13
	PG-(44:12)	$C_{50}H_{75}O_{10}P$	$[M-H]^-$	865.5025	865.5033	0.92
	PI(38:6)	$C_{47}H_{79}O_{13}P$	$[M-H]^-$	881.5186	881.5195	1.02

(continued)

Species	Name	Formula	Ion type	Theoretical *m/z*	Measured *m/z*	Relative error(ppm)	Species	Name	Formula	Ion type	Theoretical *m/z*	Measured *m/z*	Relative error(ppm)
**PI**	PI(36:4)	$C_{45}H_{79}O_{13}P$	$[M-H]^-$	857.5186	857.5198	1.40	**Others**	Ascorbic acid*	$C_6H_8O_6$	$[M-H]^-$	175.0248	175.0241	-4.00
	PI(38:5)	$C_{47}H_{81}O_{13}P$	$[M-H]^-$	883.5342	883.5346	0.45		pantothenic acid*	$C_9H_{17}NO_5$	$[M-H]^-$	218.1034	218.1027	-3.21
	PI(38:4)*	$C_{47}H_{83}O_{13}P$	$[M-H]^-$	885.5499	885.5509	1.13		phosphoric acid*	H_3O_4P	$[M-H]^-$	96.9696	96.9692	-4.13
	PI(40:6)	$C_{49}H_{83}O_{13}P$	$[M-H]^-$	909.5499	909.5509	1.10		Sarcosine	$C_3H_7NO_2$	$[M-H]^-$	88.0404	88.0400	-4.54
	PI(40:4)	$C_{49}H_{87}O_{13}P$	$[M-H]^-$	913.5812	913.5815	0.33		Gluconic acid*	$C_4H_{12}O_7$	$[M-H]^-$	195.051	195.0504	-3.08
**Sulfatide**	C22-OH Sulfatide	$C_{46}H_{89}NO_{12}S$	$[M-H]^-$	878.6032	878.6039	0.80		Glucose*	$C_6H_{12}O_6$	$[M-H]^-$	179.0561	179.0554	-3.91
										$[M+Cl]^-$	215.0328	215.0324	-1.86
	C24:1-OH Sulfatide	$C_{48}H_{91}NO_{12}S$	$[M-H]^-$	904.6189	904.6198	0.99		Aminobutyric acid*	$C_4H_9NO_2$	$[M-H]^-$	102.0561	102.0556	-4.90
	C24-OH Sulfatide	$C_{48}H_{93}NO_{12}S$	$[M-H]^-$	906.6346	906.6356	1.10		cholesterol sulfate*	$C_{27}H_{46}O_4S$	$[M-H]^-$	465.3044	465.305	1.29
**Others**	Glyceryl-Phosphoryl-ethanolamine*	$C_5H_{14}NO_6P$	$[M-H]^-$	214.0486	214.0476	-4.67		Glucose-phosphate*	$C_6H_{13}O_9P$	$[M-H]^-$	259.0224	259.0223	-0.39
	Phosphorylethanolamin*	$C_2H_8NO_4P$	$[M-H]^-$	140.0118	140.0111	-4.71		Glycerol-phosphate*	$C_4H_9O_6P$	$[M-H]^-$	171.0064	171.0057	-4.09

The metabolites identified by HRMS/MS were marked with *.
